# Pathogenesis of Anemia in Canine Babesiosis: Possible Contribution of Pro-Inflammatory Cytokines and Chemokines—A Review

**DOI:** 10.3390/pathogens12020166

**Published:** 2023-01-20

**Authors:** Wojciech Zygner, Olga Gójska-Zygner, Luke J. Norbury

**Affiliations:** 1 Division of Parasitology and Parasitic Diseases, Department of Preclinical Sciences, Institute of Veterinary Medicine, Warsaw University of Life Sciences—SGGW, Ciszewskiego 8, 02-786 Warsaw, Poland; 2 Labros Veterinary Clinic, Św. Bonifacego 92, 02-940 Warsaw, Poland; 3 Department of Biosciences and Food Technology, School of Science, STEM College, RMIT University, Bundoora, VIC 3083, Australia

**Keywords:** anemia, *Babesia*, canine babesiosis, chemokines, cytokines

## Abstract

Canine babesiosis is a tick-borne protozoan disease caused by intraerythrocytic parasites of the genus *Babesia*. The infection may lead to anemia in infected dogs. However, anemia is not directly caused by the pathogen. The parasite’s developmental stages only have a marginal role in contributing to a decreased red blood cell (RBC) count. The main cause of anemia in affected dogs is the immune response to the infection. This response includes antibody production, erythrophagocytosis, oxidative damage of RBCs, complement activation, and antibody-dependent cellular cytotoxicity. Moreover, both infected and uninfected erythrocytes are retained in the spleen and sequestered in micro-vessels. All these actions are driven by pro-inflammatory cytokines and chemokines, especially IFN-γ, TNF-α, IL-6, and IL-8. Additionally, imbalance between the actions of pro- and anti-inflammatory cytokines plays a role in patho-mechanisms leading to anemia in canine babesiosis. This article is a review of the studies on the pathogenesis of anemia in canine babesiosis and related diseases, such as bovine or murine babesiosis and human or murine malaria, and the role of pro-inflammatory cytokines and chemokines in the mechanisms leading to anemia in infected dogs.

## 1. Introduction

Canine babesiosis is a tick-transmitted disease caused by infection with intraerythrocytic protozoan parasites of the genus *Babesia*. *Babesia* belong to the order Piroplasmida, phylum Apicomplexa [1]. There are seven *Babesia* species infecting dogs: *B. canis*, *B. vogeli*, *B. rossi*, *B. vulpes*, *B. gibsoni*, *B. conradae,* and unnamed *Babesia* sp. “Coco” [1,2,3,4,5]. Some authors use the name *Babesia coco* as a formal species name [6]. Among these species, *B. canis*, *B. vogeli*, *B. rossi*, and *B. coco* are classified as ‘large’ *Babesia*, while *B. gibsoni*, *B. vulpes,* and *B. conradae* are ‘small’ *Babesia* species [7]. Severity of infection may be subclinical, mild, moderate, or severe, with a fatal outcome, and this depends on the species of pathogen and the immune response. Severe babesiosis, such as human malaria, is considered not only a parasitic disease but also an immune-mediated disease with an excessive inflammatory response, especially overproduction of pro-inflammatory cytokines and chemokines. This is one of the reasons that these diseases, both caused by intraerythrocytic protozoan parasites, are considered similar. Moreover, sequestration of red blood cells (RBC) in micro-vessels leading to vascular obstruction occurs during both *Plasmodium* and *Babesia* infections. These two mechanisms lead to similar pathologies in human malaria and in babesiosis in various mammalian species, including canine babesiosis [8,9,10]. Due to the similarities between these two infections, human babesiosis is sometimes even named “Malaria of the North” [11].

Both parasitoses may cause anemia. This pathology is the second-most prevalent hematological disorder in these diseases, after thrombocytopenia. Leukopenia is another disorder observed during these infections. However, leukocytosis has been observed in some patients with malaria [8,12,13,14,15,16,17]. Complications such as disseminated intravascular coagulation, kidney injury, pancreatitis, hepatopathy, cardiac disorders, cerebral babesiosis/malaria, and acute respiratory distress syndrome have been observed in both diseases [2,8,18,19,20,21]. These protozoan infections may lead to systemic inflammatory response syndrome, multiple organ dysfunction syndrome, and shock; consequently, these infections are considered as conditions similar to sepsis, with some authors considering them as protozoan sepses. Pro-inflammatory cytokines and chemokines, particularly tumor necrosis factor alpha (TNF-α), interferon gamma (IFN-γ), monocyte chemoattractant protein 1 (MCP-1, also known as CCL2), keratinocyte-derived chemokine (KC, also known as CXCL1)-like, interferon gamma-induced protein 10 (IP-10, also known as CXCL10), interleukin 6 (IL-6), IL-8 (also known as CXCL8), IL-12, IL-18, granulocyte-macrophage colony-stimulating factor (GM-CSF, also known as CSF-2), and high-mobility group box-1 protein (HMGB-1), play a role in the development of many of these complications. Moreover, insufficient and/or delayed production of anti-inflammatory cytokines, such as IL-4 and IL-10, also contributes to the pathogenesis of both diseases, including hematological changes [2,22,23,24,25,26,27,28,29,30].

## 2. Anemia

Anemia during canine babesiosis is observed in 20% to over 90% of infected dogs [31,32,33,34,35,36,37,38,39]. The most severe and prevalent anemia occurs in dogs infected with *B. rossi* and *B. vulpes* [31,32,38]. Decreased hematocrit is a prognostic marker in *B. canis*-infected dogs and is significantly lower in non-survivors in comparison to survivors [40]. Anemia during canine babesiosis results from intra- and extra-vascular hemolysis. Extravascular hemolysis is caused by spleen and liver phagocytes, whereas intravascular hemolysis results from both the lifecycle of the parasite (merogony) and immune-mediated lysis of RBCs [7,8]. During both malaria and babesiosis, the level of parasitemia does not correlate with the severity of anemia [8,41]. Price et al. [42] estimated that over 90% of destroyed RBCs in humans infected with *Plasmodium falciparum* were not invaded by the parasite. In another study, a mathematical model of Jakeman et al. [43] showed that in patients with malaria, 8.5 uninfected erythrocytes were destroyed in addition to 1 infected RBC. Moreover, this model showed that dyserythropoiesis has a marginal role in the development of anemia in malaria. A lack of association between the level of parasitemia and anemia is also observed in dogs infected with *B. rossi*, *B. canis,* and *B. gibsoni* [8,35,44,45], and as in malaria, dyserythropoiesis in canine babesiosis has, if any, an insignificant role in the development of anemia [8,36]. This indicates that direct destruction of infected RBCs by the pathogen is not the main cause of anemia in dogs infected with *Babesia* spp.

However, it should be mentioned that a correlation between the level of parasitemia and anemia has been observed in humans infected with *B. microti* [46]. Moreover, asymptomatic infections may occur in humans with low *B. microti* parasitemia [47]. In bovine theileriosis, a disease of cattle similar to babesiosis, the level of parasitemia has been shown to correlate with the severity of anemia, although immunological mechanisms also contribute to its development [48]. Similarly, in dogs experimentally infected with the other piroplasmid pathogen, *Rangelia vitalii*, hematocrit was lowest at peak parasitemia, before clinical signs of hemorrhage had manifested [49]. However, changes typical for immune-mediated anemia in canine rangeliosis have also been observed [50].

The lack of association between the level of parasitemia and the severity of anemia in canine babesiosis indicates that the immune response during infection contributes to decreasing the number of RBCs. Phagocytosis, oxidative damage of erythrocytes, antibodies, and the complement system participate in the response to infection. These responses lead to both killing of the parasite and anemia, with other complications of the disease occurring in more severe cases. Moreover, besides immune-mediated hemolytic anemia, sequestration of erythrocytes in microvasculature and splenic retention of RBCs may also negatively impact the RBC count in infected dogs [8,39,51,52].

## 3. Phagocytosis

Phagocytosis is an important mechanism of innate immunity which is used to remove pathogens, cell debris, foreign substances, and apoptotic cells. These targets are recognized by various receptors on phagocytic cells. The process leads to phagosome and further phagolysosome formation, in which microorganisms and other targets are killed and degraded by reactive oxygen species, hypochlorous acid, and various hydrolytic enzymes [53].

**Figure 1:**
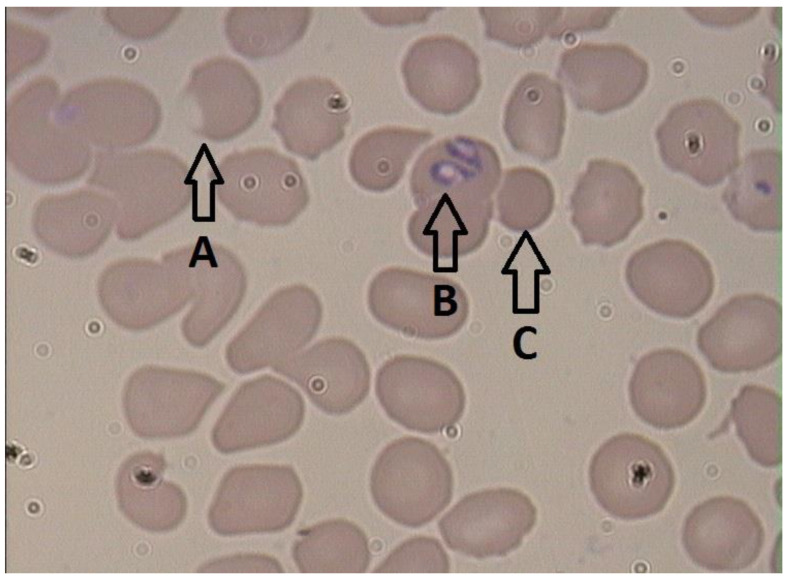
Blood smear from a dog infected with *B. canis*: A—eccentrocyte, B—*Babesia* merozoites, C—spherocyte.

In 1990, Murase and Maede [54] showed that macrophages obtained from splenectomized dogs infected with *B. gibsoni* had increased erythrophagocytic activity. This phenomenon was not observed in macrophages obtained from dogs with onion-induced hemolysis [54]. A further study showed that following in vitro culture of *B. gibsoni* with erythrocytes, both infected and uninfected RBCs were more susceptible to phagocytosis by macrophages obtained from healthy dogs [51]. These results suggest involvement of the parasite in macrophage activation. A study of *B. rossi* showed an increased proportion of both splenic and bone marrow-derived macrophages in the spleens of infected dogs in comparison to the spleens of healthy dogs [55]. Moreover, in dogs infected with *B. gibsoni* or *B. canis,* spherocytosis has been observed (Figure 1) [35,56,57]. This pathology is typical for immune-mediated hemolytic anemia and results from partial phagocytosis of erythrocytes by macrophages [58]. These observations showed activation of macrophages in *Babesia*-infected dogs and highlight the contribution of macrophages to the development of anemia in canine babesiosis.

Similar changes have been observed during other apicomplexan infections. In mice experimentally infected with *B. microti,* increased numbers of macrophages were observed in spleens [59]. In another study on *B. microti*, spherocytes were demonstrated in infected mice [60]. The same changes also occur in malaria, bovine theileriosis, and canine rangeliosis, and result from increased phagocytosis of both parasitized and non-parasitized RBCs [50,61,62,63,64].

In addition to macrophages, increased phagocytic activity of neutrophils has been observed in cattle infected with *B. bovis* [65]. According to Bloch et al. [66], neutrophils contribute to the phagocytosis of *Babesia*-infected RBCs in the marginal zone of the spleen. However, according to Court et al. [65], this granulocytic phagocytosis of RBCs occurs in the peripheral circulation rather than in the spleen. The latter is in agreement with observations in malaria patients, with neutropenia caused by intravascular redistribution of neutrophils leading to enlargement of the marginal granulocyte pool [67]. Moreover, Henning et al. [55] observed decreased numbers of neutrophils in the spleens of dogs infected with *B. rossi*. This may confirm that the action of neutrophils during both malaria and babesiosis infections occurs peripherally, rather than in the spleen. These granulocytes may be engaged in phagocytosis of RBCs sequestered in small vessels, where sequestration is caused by adhesion of parasitized erythrocytes to endothelial cells. This phenomenon has been observed in both malaria and babesiosis and is discussed in detail in a later section [68]. Neutrophilic infiltrations have been observed in various organs in both diseases [69,70,71,72]. However, the number of capillary neutrophils is relatively low in most cases, except during chronic human placental malaria and murine cerebral malaria [69].

As mentioned in the introduction, TNF-α and IFN-γ play a significant role in the pathogenesis of babesiosis. TNF-α induces gene expression of CXCL8 and CXCL1, and together with IFN-γ induces differentiation of monocytes to macrophages; in addition, it stimulates the synthesis of nitric oxide (NO) by inducible nitric oxide synthase (iNOS) in these phagocytic cells [73,74,75,76]. Moreover, CCL2 also contributes to the classical activation of macrophages and may play a role in macrophage recruitment to the spleen. This chemokine, previously considered a chemoattractant only for monocytes, may also attract other cells, such as natural killer (NK) cells, T and B lymphocytes, basophils, dendritic cells, and neutrophils [77,78]. Increased CCL2 levels have been observed in malaria and canine babesiosis caused by *B. rossi* and *B. canis* [28,30]. Higher serum levels of CCL2 have been associated with *B. rossi* and *B. canis* infection severity, and with fatal outcomes from *B. rossi* infections [28,30,79]. However, the reported negative correlation between the serum concentration of CCL2 and hematocrit was not found to be statistically significant during *B. rossi* infection [79]. Production of this chemokine is induced by platelet-derived growth factor, which is secreted by activated platelets. CCL2 is produced and released by various cells, such as endothelial cells, fibroblasts, and macrophages, and attracts monocytes to the site of pathogen infection. This has been confirmed during *Leishmania major* infection [80]. Therefore, as *Babesia*-infected erythrocytes adhere to endothelial cells in small blood vessels while circulating infected RBCs flow to the spleen, CCL2, a chemoattractant for both monocytes and neutrophils, may consequently play a role in both splenic and peripheral phagocytosis of RBCs. The association between the concentration of CCL2 and the severity of infection also indicates classical monocyte activation in severe cases of canine babesiosis.

Another cytokine, CSF-2, is also involved in classical activation of macrophages [81]. Increased serum levels of this cytokine have been observed during canine babesiosis caused by *B. rossi* and *B. canis* at the beginning of the disease [28,30]. Moreover, Galán et al. [30] demonstrated higher concentrations of CSF-2 in complicated cases in comparison to uncomplicated cases in dogs infected with *B. canis* on the first day of disease (but not on the seventh day) and showed a positive correlation between CSF-2 concentration and eosinophil count in affected animals. This correlation may result from the fact that eosinophils are cells that produce CSF-2. However, other cells such as fibroblasts, endothelial cells, neutrophils, T lymphocytes, and macrophages also produce this cytokine. Expression of CSF-2 is induced by TNF-α, but IFN-γ and the anti-inflammatory cytokine IL-10 suppress it [81]. Increased production of IFN-γ over the course of babesiosis may explain the lack of difference in CSF-2 levels between dogs with complicated and uncomplicated disease by the seventh day of infection.

During human malaria, the level of CSF-2 increases, but is not associated with disease severity [82]. However, CSF-2-deficient mice infected with *P. chabaudi* have been shown to have higher peak parasitemia, higher mortality, impaired splenomegaly, and a lower leukocyte count in comparison to wild-type-infected mice, despite equivalent levels of anemia in both groups [83]. That study showed involvement of CSF-2 in activation of granulocyte-macrophage hematopoiesis and resistance to *P. chabaudi* infection [83]. An explanation for the lack of differences in the levels of anemia in infected mice may stem from the action of the malarial pigment hemozoin, which impairs CSF-2 receptor expression and function [84]. However, *Babesia* spp. do not produce this pigment, and consequently do not impair the CSF-2 receptor [60]. CSF-2 contributes to the recruitment of neutrophils and monocytes to the site of infection, the mobilization of monocytes from bone marrow, and stimulates production of CCL2 by neutrophils and macrophages [81,85]; therefore, this cytokine in combination with CCL2 may be involved in phagocytosis at the acute phase of the infection.

Classical stimulation of monocytes by *Babesia* merozoites in the presence of TNF-α and IFN-γ is inhibited by both IL-4 and IL-10 [86]. These two interleukins play a role in regulation of inflammatory processes: IL-4 leads to alternative activation of macrophages, while IL-10 decreases the activity of Th1 cells, macrophages, and NK cells [87,88]. Leisewitz et al. [79] showed that during canine babesiosis caused by *B. rossi*, the IL-10 serum concentration was lower in severe cases in comparison to uncomplicated cases of the disease. In the same study, the first group also had significantly lower hematocrit. While there was no correlation between hematocrit and IL-10 in infected dogs, hematocrit was strongly positively correlated with IL-8 in these dogs [79]. Alternatively, negative correlations between IL-10 and both hematocrit and the number of RBCs have been observed in *B. canis*-infected dogs, but only in uncomplicated babesiosis [30]. Increased levels of IL-8 have been reported in canine babesiosis caused by *B. canis* in comparison to healthy dogs, but with no differences between medians in complicated and uncomplicated cases [30,89]. Serum concentrations of IL-8 also show increases during sepsis and malaria [90,91]. Similar changes to the concentration of CXCL1-like have been observed during *B. canis* infections [30,89]. This is not surprising as this chemokine is considered a functional homolog of IL-8 [92]. The concentration of CXCL1-like, as per IL-8, is associated with the severity of anemia in *B. canis* infections [89]. Surprisingly, IL-8 levels have previously been reported as lower in dogs naturally infected with *B. rossi* in comparison to healthy dogs [28]. A possible explanation for this observation was missing the peak chemokine concentration, and later studies on experimental *B. rossi* infections have confirmed this supposition [27,93]. In dogs infected with *B. gibsoni*, both an increase and a decrease in IL-8 levels have been observed [94], while a transient increase of CXCL1-like has been reported, similar to IL-8, in *B. canis* infection. However, the research on *B. gibsoni* infection consisted of experimental infection of only two dogs.

Further studies of pro-inflammatory cytokines and chemokines during *B. rossi* natural infections have shown negative correlations between IL-8 concentration and the severity of disease and fatal outcomes [79]. Leisewitz et al. [79] speculated on the possibility of late sample collection as a reason for these results. As mentioned earlier, experimental studies on *B. rossi* infections showed a transient increase of IL-8 four days after infection, when the level of parasitemia was high. The level of IL-8 subsequently decreases, but not to low values [27,93]. The same studies showed similar transient increases in CXCL1-like concentration four days after infection, followed by decrease to a low level [27,93]. According to Smith et al. [93], increased production of IL-10 may be a possible explanation for a decrease in IL-8. This speculation is supported by the results of Galán et al. [30], who observed a negative correlation between IL-8 and hematocrit and increased levels of this chemokine even on the seventh day of the disease. Decreased IL-10 serum concentration on the seventh day of babesiosis caused by *B. canis* was also observed [30]. Thus, it seems possible that immunosuppressive IL-10, or rather its insufficient production, may play a role in the development of anemia in canine babesiosis.

IL-8 and CXCL1-like are chemokines that induce chemotaxis, and activate and stimulate phagocytosis by neutrophils [92,95,96]. These granulocytes participate in phagocytosis of *Plasmodium*-infected RBCs, and the concentration of IL-8 has been positively correlated with the level of parasitemia in *P. falciparum* malaria [97,98]. In *B. rossi*-infected dogs, IL-8 is positively correlated, and IL-10 is negatively correlated, with the number of segmented neutrophils and lymphocytes [28]. During *B. canis* infection, Galán et al. [30] showed a positive correlation between IL-8 and the number of band neutrophils. An increased band neutrophil count has also been observed together with segmented neutropenia in both canine babesiosis and human malaria [36,67]. This probably results from the intravascular shift of neutrophils from the circulation pool to the marginal pool. Moreover, as mentioned earlier, increased phagocytic activity of neutrophils has also been observed in bovine babesiosis caused by *B. bovis* [65,67]. The repeated associations between anemia and cytokines and chemokines, such as IL-10, IL-8, and CXCL1-like, indicate that increased production of IL-8 and CXCL1-like, and/or insufficient production of IL-10, can have an influence on phagocytic anemia in canine babesiosis.

Another cytokine which may be involved in erythrophagocytosis is IL-18 [99]. Serum levels of this cytokine have been determined in dogs infected with *B. canis* and *B. rossi* [28,30]. Increased levels were observed only in the acute phase of infection [28]. In human malaria, an elevated level of this interleukin has been associated with the severity of the disease [100]. Moreover, a high serum concentration of IL-18 was observed in humans with secondary hemophagocytic syndrome and correlated with the severity of anemia in affected individuals [101]. As this cytokine is elevated only at the beginning of canine babesiosis [28], it seems probable that further control of infection depends on other mechanisms involving IFN-γ and TNF-α, which are triggered by IL-18 together with IL-12 [99,102]. According to Suarez et al. [103], RBC destruction in bovine babesiosis results from early induction of IL-18, IL-12, IFN-γ, and TNF-α secretions and late action of IL-10.

It is also worth mentioning that neutrophils together with monocytes are the main source of CXCL10 in the spleen during murine malaria [104]. This chemokine is connected to the severity of malaria, including cerebral malaria and hemolysis leading to severe anemia [105]. However, in canine babesiosis, Atkinson et al. [27] did not observe significant changes to CXCL10 in dogs experimentally infected with *B. rossi* in comparison to healthy animals; however, the study was on a very small group of young (6-month-old) beagles [27]. Decker et al. [106] and Hayney et al. [107] showed a positive association between CXCL10 concentration and increasing age in humans (both in children and adults); thus, younger dogs may have a lower concentration of this chemokine. CXCL10 is important in the recruitment of granulocytes and macrophages and their phagocytic function [108]. Moreover, CXCL10 is associated with warm autoimmune hemolytic anemia in humans, and this type of hemolysis has been recognized in humans after babesiosis [109,110]. Thus, a role for CXCL10 in *Babesia*-infected dogs cannot be excluded.

It seems probable that the action of neutrophils during canine babesiosis, as in malaria, may have an influence on the severity of anemia caused by phagocytosis. However, CXCL10′s involvement in hemolysis in *Babesia*-infected dogs is unclear and requires further study, especially in adult dogs.

## 4. Oxidative Damage

Anemia during canine babesiosis may also result from oxidative damage to erythrocytes. This is caused by the release of reactive oxygen species, which may be an intra- or extra-cellular phenomenon, as a part of phagocytosis or independent of it [45,111].

Oxidative damage of RBCs has been observed in dogs infected with *B. gibsoni* [51,112]. These studies observed increased serum concentrations of malondialdehyde, which is the end-product of lipid peroxidation, and increased production of superoxide in RBCs cultured with *B. gibsoni*. Moreover, Murase et al. [51] demonstrated an increased concentration of methemoglobin in a culture of erythrocytes incubated with *B. gibsoni*, while Otsuka et al. [112] revealed that lipid peroxidation was higher in infected compared to non-infected erythrocytes. These results showed that oxidative damage to RBCs is caused by the parasite or by parasitized cells. However, Morita et al. [113] showed that the ratio of methemoglobin to total hemoglobin concentration was similar in splenectomized and intact dogs infected with *B. gibsoni*, despite parasitemia being three times higher in splenectomized animals. Otsuka et al. [45] observed increased lipid peroxidation of erythrocytes obtained from healthy dogs when incubated with macrophages derived from peripheral monocytes from *B. gibsoni*-infected dogs in 2002. These results indicate that macrophages and the spleen contribute to the oxidative damage of RBCs in babesiosis caused by *B. gibsoni*.

The serum malondialdehyde concentration has also been determined in dogs infected with *B. canis* [114], with a higher concentration of malondialdehyde observed in infected dogs compared to healthy animals. However, no differences in malondialdehyde concentration were reported between dogs with complicated and uncomplicated disease, or between dogs with various severities of anemia [114]. In 2002, Jacobson et al. [115] published the results of a study on nitric oxide metabolites in dogs infected with *B. rossi*. The highest concentration of reactive nitrogen intermediates was detected in infected dogs with severe anemia. However, there was a lack of association between increased reactive nitric intermediates and the severity of the disease [115]. In 2009, Carli et al. [39] published the results of their study on the pathogenesis of anemia in canine babesiosis caused by *B. canis* and *B. vogeli*. The authors detected eccentrocytosis in 33% of dogs infected with *B. canis*, but not in dogs infected with *B. vogeli*. This pathology is typical of oxidative damage to RBCs, and results from peroxidation of cytoskeletal elements or cell membranes leading to shifting of the cytoplasm together with hemoglobin and leaving a pale area on one side of the margin of the erythrocyte (Figure 1) [39,116]. Eccentrocytosis showed that there is oxidative damage to RBCs during *B. canis* infection. However, the results of other studies on large *Babesia* [114,115] suggest that oxidative damage of erythrocytes may contribute less to the development of anemia in other canine babesiosis than in the disease caused by *B. gibsoni*.

Other studies on both small and large *Babesia* spp. in dogs have demonstrated increased activation of antioxidant defense in affected animals. In dogs naturally infected with *B. gibsoni*, the activities of erythrocytic antioxidant enzymes (catalase and superoxide dismutase) were higher than in control healthy dogs [117]. Moreover, higher levels of lipid peroxides in RBCs (expressed as a number of nanomoles of malondialdehyde per one milligram of hemoglobin), and decreased concentrations of copper, zinc, and iron in blood samples, were present in infected compared to uninfected animals [117]. Comparable results were observed in dogs with uncomplicated babesiosis caused by *B. canis*, with increased activities of superoxide dismutase and catalase and decreased concentrations of zinc and copper in the blood of affected dogs [118]. *B. canis*-infected animals also had pale mucosal membranes and dark urine, indicating methemoglobinuria, reflecting acute intravascular hemolysis [118]. The results of these studies on antioxidant status in canine babesiosis indicates an association between oxidative stress and anemia. However, during *B. canis* infection, no association between the severity of anemia and the activities of catalase or superoxide dismutase were shown [118].

Another study on *B. canis* infection and oxidative stress [119] conversely showed opposing results to those obtained by Chaudhuri et al. [117] and Teodorowski et al. [118]. Decreased activities of superoxide dismutase, catalase, and glutathione peroxidase in the blood of infected animals, and decreased serum concentration of total antioxidant status (represents total contribution of various antioxidants), were observed in infected dogs in comparison to healthy dogs [119]. Crnogaj et al. [119] speculated on the discrepancies between the study results on *B. canis* and *B. gibsoni* [117] resulted from differences between the parasite species and kits used in the studies [119]. However, Teodorowski et al. [118] and Crnogaj et al. [119] worked with the same *Babesia* species and used the same kit for determination of superoxide dismutase activity. Teodorowski et al. [118] speculated that the discrepancies might result from the stage of the disease, suggesting increased erythrocytic antioxidant enzyme activities in the early stage of disease, and this explanation seems logical. Crnogaj et al. [119] observed lower antioxidant enzyme activities in complicated babesiosis in comparison to uncomplicated disease, with the lowest enzyme activities in *B. canis*-infected dogs with multiple organ dysfunction syndrome. However, even dogs with uncomplicated babesiosis had significantly lower activities of superoxide dismutase and catalase than healthy dogs [119]. The study of Crnogaj et al. [119] showed another interesting result. In the comparison between anemic and non-anemic infected dogs, significant differences were shown only in the activity of superoxide dismutase and the serum concentration of malondialdehyde [119]. Superoxide dismutase activity was lower in anemic dogs with babesiosis. Decreased activity of this enzyme in anemic dogs, and the lower activities of the other antioxidant enzymes in infected dogs, may result from their consumption in oxidative processes over the course of the disease. This supposition is supported by another result from the study on the increased concentration of malondialdehyde in infected dogs, where a higher concentration of malondialdehyde in anemic dogs shows the contribution of oxidative processes in the development of anemia in canine babesiosis [119]. Moreover, Crnogaj et al. [119] observed a negative correlation between hematocrit and malondialdehyde, and positive correlations between hematocrit and the activities of both superoxide dismutase and glutathione peroxidase in infected dogs. These results confirmed oxidative damage of RBCs and consumption of erythrocytic antioxidant enzymes during the progression of canine babesiosis. However, the lack of difference in malondialdehyde concentration between dogs with various severities of anemia, despite the negative correlation with hematocrit, indicates that other mechanisms contribute to RBC destruction over the course of canine babesiosis.

Similar results indicating oxidative damage to RBCs have been observed during equine babesiosis and theileriosis, caprine babesiosis, bovine babesiosis and theileriosis, ovine babesiosis, and human malaria [120,121,122,123,124,125,126,127]. In these studies, akin to the study of Crnogaj et al. [119] on antioxidant status in canine babesiosis, the activities of erythrocytic antioxidant enzymes were reduced in infected humans and animals in comparison to control groups.

In 2015, two studies on platelet activation in *B. rossi*-infected dogs were published. In one study, infected dogs had an increased percentage of platelet–monocyte aggregates [128]. The second work showed an increased number of large-activated platelets over the course of the disease [129]. Subsequently, Kho et al. [15] demonstrated that platelets were involved in the killing of erythrocytic stages of all four major human *Plasmodium* species. In 2021, Annarapu et al. [130] demonstrated that extracellular hemoglobin leads to platelet activation. This results in the production of mitochondrial reactive oxygen species and platelet degranulation. Platelet factor-4 (PF-4, also known as CXCL4) accumulates in infected erythrocytes and is the chemokine involved in platelet activation, inducing production of mitochondrial reactive oxygen species [130]. These results indicate that platelets may be involved in oxidative damage of RBCs in canine babesiosis. Interestingly, CXCL4 has been shown to be associated with cerebral malaria in both humans and mice. However, cerebral malaria and cerebral babesiosis are associated with sequestration of parasitized RBCs in cerebral capillary vessels [131,132,133,134], and platelets play a role in the formation of micro-aggregates of parasitized erythrocytes in micro-vessels [135]. Nevertheless, further studies on the activation of thrombocytes, CXCL4, and oxidative stress in canine babesiosis could explain if this chemokine contributes to anemia over the course of an infection. According to the authors’ knowledge, the level of CXCL4 has not been determined in *Babesia*-infected dogs.

TNF-α appears to be the most important cytokine with a role in oxidative damage of RBCs during canine babesiosis. As with human malaria, increased concentrations of this cytokine have been observed in canine, bovine, murine, and human babesiosis [22,28,59,79,136,137,138]. As mentioned earlier, TNF-α and INF-γ induce the synthesis of nitric oxide (NO) by inducible nitric oxide synthase (iNOS) [73]. Moreover, IL-8 activates neutrophils and potentiates the oxidative burst induced by microbial stimuli such as formyl-methionyl-leucyl-phenylalanine [139]. Further, stimulation of neutrophils by HMGB-1 activates their adhesive and migratory functions, and production of reactive oxygen species [140], and this cytokine has also been associated with oxidative stress [141]. Oxidative burst results from activation of nicotinamide adenine dinucleotide phosphate (NADPH) oxidase. This enzyme is also induced by phagocytosis. NADPH oxidase contributes to neutrophil apoptosis, the formation of neutrophil extracellular traps (NETs), and converts molecules of oxygen to superoxide anions [142]. Extracellular superoxide and nitric oxide form peroxynitrite, which is involved in the killing of the parasite, but it also leads to cytotoxicity by direct peroxidation of lipids in cell membranes or the formation of reactive hydroxyl radical and nitrogen dioxide [143,144].

Court et al. [65] showed that monocytes display decreased phagocytic activity but increased oxidative burst in bovine babesiosis caused by *B. bovis*. This may indicate the involvement of monocytes in oxidative damage of erythrocytes during babesiosis. As mentioned earlier, activation of monocytes by IFN-γ results in classical activation of macrophages, leading to the release of pro-inflammatory cytokines such as IL-6 and TNF-α [145], and increased concentration of IFN-γ has been observed during both babesiosis and malaria [59,138,146]. In malaria patients, an increased level of IFN-γ was shown to be associated with the severity of anemia; however, no association between TNF-α concentration and anemia was shown in the same study [147]. The authors postulated that this observation could be explained by the fact that the patients probably had prolonged or subacute courses of the infection, and consequently, their pro-inflammatory cytokine levels were lower [147]. Subsequently, other studies on human malaria showed an association between the severity of anemia and the serum concentration of TNF-α [148], or that TNF-α was associated with the severe course of the disease [149]. In the latter research, an increased TNF-α to IL-10 ratio was associated with severe malaria, defined as more than one of severe anemia, coma, or respiratory distress [149]. Thus, not only increased production of TNF-α, but also an imbalance between TNF-α and IL-10 production, influences oxidative damage of RBCs. Moreover, as mentioned earlier, IL-10 as well as IL-4 cause inhibition of IFN-γ- and TNF-α-dependent nitric oxide production by mononuclear phagocytes exposed to merozoites isolated from *B. bovis*-infected erythrocytes [86]. However, according to the authors’ knowledge, IL-4 levels have not been evaluated in dogs with babesiosis.

Studies on canine babesiosis have shown increased serum IL-6 concentrations in dogs infected with *B. canis*, *B. rossi,* and *B. gibsoni* [28,79,94,150]. In *B. rossi*-infected dogs, a higher IL-6 concentration was associated with the severity of disease and fatal outcomes [79]. However, IL-6 was positively correlated with hematocrit in affected animals in that study [79]. This result might indicate a lack of influence by IL-6 on the development of anemia in canine babesiosis. In human malaria, as in canine babesiosis, the IL-6 serum concentration has been found to be increased and associated with the severity of disease [151]. Further, elevated IL-6 levels have also been associated with the severity of anemia in humans infected with *P. vivax* and *P. falciparum* [148]. Moreover, other studies on various diseases have shown the influence of IL-6 on the development of oxidative stress [152,153,154]. For instance, Petrushevska et al. [152] observed correlations between various pro-inflammatory cytokines and markers of oxidative stress in patients with COVID-19, with IL-6 showing the strongest correlations. However, some studies have shown opposite actions for IL-6 and proven its protective role in oxidative stress [155,156,157]. Moreover, other authors have shown that an elevated IL-6 concentration is a consequence of increased reactive oxygen species [158,159]. IL-6 levels appear to correlate with oxidative stress during infections. It is currently unclear whether IL-6 has a role in promoting this process or facilitates unrelated or protective mechanisms, and as such its role in the development of anemia through oxidative damage of RBCs during canine babesiosis is unclear and further investigation is required.

Another cytokine that contributes to the production of nitric oxide and reactive oxygen species is IL-18 [99]. This cytokine, especially in synergy with IL-12, induces production of mitochondrial reactive oxygen species [102]. The serum level of IL-18 is increased in the acute phase of canine babesiosis [28]. It seems probable that in this disease, as in bovine babesiosis, early increased production of IL-18 and IL-12 stimulates the release of IFN-γ and TNF-α, leading to activation of iNOS and NO production, which together with delayed secretion of IL-10 causes oxidative damage to erythrocytes. This response, required for inhibition of parasite replication, together with late IL-10 production, seems to be one of the main causes of immune-mediated destruction of erythrocytes [103,160].

It is also worth mentioning that secretion of IFN-γ when stimulated by IL-18 and IL-12 requires mitochondrial reactive oxygen species as redox signaling molecules [102]. Thus, increased levels of IL-18, IFN-γ, and TNF-α, together with eccentrocytosis observed during canine babesiosis, suggest the contribution of these cytokines in oxidative damage of RBCs in infected animals. However, further studies on the involvement of these cytokines in oxidative stress and anemia during the disease are required. According to the authors’ knowledge, the serum concentration of IL-12 has not been evaluated during canine babesiosis.

It should be emphasized that cytokines such as IL-18, IL-12, IFN-γ, and TNF-α play an important role in clearance of the parasite by stimulation of reactive oxygen and nitrogen intermediates [99,160,161]. This is supported by cases where concurrent treatments that reduce the levels or effectiveness of these cytokines coincide with high parasite burdens, such as: a 57-year-old woman with Crohn’s disease treated with infliximab (a TNF-α-neutralizing monoclonal antibody) with a high level of parasitemia over the course of babesiosis, or a 67-year-old man with severe *B. microti* infection, who owing to rheumatoid arthritis was being treated with etanercept (a competitive blocker of TNF-α and TNF-β receptors) [162,163]. A further example of the significant role of these cytokines in *Babesia* control comes from an in vitro study which revealed increased production of IL-1β, IL-12, and TNF-α by macrophages activated by *B. bovis*, and reduced parasite viability when the macrophages were incubated with *B. bovis*-infected RBCs [160]. Moreover, Aguilar-Delfín et al. [164] showed that infection of mice with *B. duncani* led to increased serum concentrations of IL-12 and IFN-γ, and that mice genetically deficient in NK cells or macrophages had higher susceptibility to infection by the parasite.

The results of the studies cited above show that oxidative stress increases during infections, and that it is a likely involved in damage to RBCs, contributing to anemia. This process is modulated through the action of a number of pro-inflammatory cytokines that promote the processes involved, including phagocytosis and other mechanisms, as detailed below.

## 5. Anti-Erythrocyte Antibodies

Antibody-mediated opsonization of RBCs leads to complement activation and binding, antibody-dependent cellular cytotoxicity, and erythrophagocytosis and oxidative damage of erythrocytes [51,54,165,166,167].

Antibodies binding to RBCs can cause hemolysis, which can lead to anemia [168]. Several reports have linked babesiosis with anti-RBC antibodies and resulting autoimmune anemia. Antibodies (mainly IgG, but sometimes also IgM) binding to RBCs have been detected in dogs infected with *B. gibsoni* and *B. vogeli*, but not during canine babesiosis caused by *B. canis* [39,169,170]. Moreover, in one study, 88% of dogs infected with *B. rossi* and with severe anemia had a positive result in the Coombs test, indicating the presence of antibodies against RBCs [31]. Anti-erythrocyte antibodies have also been detected during both murine babesiosis and malaria, caused by infections with *B. rodhaini* and *P. berghei*, respectively [171,172]. Similarly, such antibodies have been detected during bovine babesiosis caused by *B. bovis* and *B. bigemina*, and in human malaria caused by *P. falciparum* and *P. vivax* [173,174,175,176]. In one study of a cohort of patients diagnosed with babesiosis caused by *B. microti*, several weeks after treatment, a positive Coombs test and hemolytic anemia was observed in six asplenic patients with no history of autoimmunity, and four patients subsequently required immunosuppressive therapy with glucocorticosteroids [110]. The successful effect of the immunosuppressive therapy together with a positive direct antiglobulin test (for both IgG and complement component 3) in the patients indicated the presence of hemolysis-causing anti-erythrocyte antibodies induced by the infection. It is worth mentioning that Narurkar et al. [177] described the case of an 81-year-old woman with babesiosis (the species was not determined) and hemolytic anemia with a positive direct antiglobulin test; however, the woman had no history of splenectomy. The authors of the case report concluded that not only asplenic but also older patients are at a higher risk of autoimmune hemolytic anemia induced by babesiosis [177].

During a *B. gibsoni* infection, anti-erythrocyte antibodies have been shown to react with cytoskeletal and transmembrane proteins [178]. In another study examining bovine babesiosis, antibodies to phosphatidylserine were detected in cattle infected with *B. bovis*, but not in cows infected with *B. bigemina* [173]. This phospholipid belongs to the inner leaflet of the erythrocyte membrane, whereas choline-containing phospholipids are located on the outer surface of the cell. This asymmetry in the cell membrane layers depends on and is maintained by ATP-driven aminophospholipid translocase/flipase, which transports phosphatidylserine to the inner layer of the cell membrane [179]. Exposure of phosphatidylserine on the outer surface is associated with the physiological aging process of RBCs. The higher and permanent exposure of phosphatidylserine on the outer surface of cell membranes is an ‘eat me’ signal for the immune system to remove such erythrocytes [180,181]. Thus, the presence of antibodies directed to phosphatidylserine in *B. bovis*-infected cattle can explain the occurrence of hemolytic anemia during infection.

Further, a study on anti-erythrocyte antibodies in cattle with babesiosis has identified autoantibodies in *B. bigemina*-infected animals [174]. The authors of the study also demonstrated that anti-idiotypic antibodies cross-react with parasitized RBCs. Similar results were previously observed in a study on malaria by Daniel-Ribeiro et al. [182], who observed cross-reaction between antigens of *P. falciparum* and idiotypes of antibodies against other plasmodial and non-plasmodial antigens.

However, according to Taboada and Lobetti [183], infected RBCs incorporate parasite antigens into their membrane surface during canine babesiosis. This induces antibodies which opsonize erythrocytes and leads to the further removal of infected cells [183]. Nevertheless, such a mechanism does not explain the lack of association between the level of parasitemia and the severity of anemia, which indicates the removal of uninfected erythrocytes and the presence of anti-erythrocyte antibodies in *Babesia*-infected dogs.

The association between the surface exposure of phosphatidylserine and anti-erythrocyte antibodies has also been studied in malaria. These studies revealed that phosphatidylserine is exposed on the surface of both *Plasmodium*-infected and uninfected RBCs, and that infection induces the production of antibodies against phosphatidylserine. This leads to binding of anti-phosphatidylserine antibodies to infected and uninfected erythrocytes [184,185]. Fernandez-Arias et al. [185] demonstrated that anti-erythrocyte antibodies recognize phosphatidylserine on the surface of uninfected erythrocytes, promoting anemia in murine malaria. The high level of anti-phosphatidylserine antibodies in malaria can lead to disregard of ‘do not eat me’ signals, such as integrin CD47, from uninfected RBCs. In such a situation, even physiologically low and transient exposure of phosphatidylserine (which is transported across the cell membrane by aminophospholipid translocase/flipase to the inner layer of the membrane) in uninfected erythrocytes is treated as an ‘eat me’ signal, despite the presence of the other ‘do not eat me’ signals such as CD47. This leads to the development of hemolytic anemia in malaria [184]. However, antibodies against other erythrocytic antigens, such as triosephosphate isomerase, band 3 (an anion exchanger protein), anticardiolipin, and spectrin, have also been detected during human malaria, and an association between autoantibodies and anemia has not always been observed [186,187].

It is possible that similar mechanisms may be involved in the production of anti-erythrocyte antibodies during canine babesiosis. Moreover, according to the authors of this review, it is probable that anti-erythrocyte antibodies participate in hemolytic anemia over the course of canine babesiosis caused by all canine *Babesia* species.

Antibodies are produced by plasma cells (mature effector B lymphocytes), and IL-7 is essential for the development of B lymphocytes [188,189]. IL-4, produced by Th2 lymphocytes, stimulates the production and secretion of IgG_1_ and IgE [188]. However, Kimata et al. [190] showed that IL-8, which is increased in both malaria and babesiosis, selectively inhibits IgE production induced by IL-4. IgG_1_ antibodies mediate antibody-dependent cellular toxicity in bovine babesiosis [191]. However, as mentioned earlier, IL-4 acts as an anti-inflammatory cytokine, promoting differentiation of naïve T cells into Th2 lymphocytes [188]. A Th2-type response is typically generated against extracellular pathogens, whereas a Th1-type response is typical against intracellular microorganisms. Both *Babesia* and *Plasmodium* are intracellular parasites [192]. However, IL-4 can also be produced during a Th1-type response by cells with active signal transducer and activator of transcription 5A (Stat5a) [192]. Xue at al. [193] showed that the spleens of mice infected with *B. microti* produced Stat5a. In another study, Djokic et al. [59] observed increased production of IL-4 in mice experimentally infected with *B. microti*. These results help explain IgG_1_ production during babesiosis; however, as the authors of the study mentioned, the increase was modest but significant [59]. During severe human malaria, the levels of IL-4 were higher in some studies and lower in others when compared to uncomplicated infections. However, a meta-analysis of the results from various studies has shown an average lower IL-4 serum concentration during severe disease in comparison to uncomplicated malaria [194].

IgG_1_ production may be also stimulated by IL-6 [195]. Production or even overproduction of IL-6 may be induced by IL-4 [196,197]. As mentioned earlier, increased levels of IL-6 have been demonstrated both during canine babesiosis and human malaria [28,79,94,148,150,151]. This cytokine is associated with a Th17 response and some autoimmune diseases. Moreover, autoimmunity is associated with activation of Th1 lymphocytes [188,189,192]. Increased production of IFN-γ is typical for a Th1 response. Production of this cytokine is stimulated by IL-12 and IL-18 [188,192]. All three of these cytokines are increased during human malaria [146,198,199], while IFN-γ and IL-18 are increased during canine babesiosis [27]. Although IL-12 levels have not been determined in *Babesia*-infected dogs, it is probable that its level would be elevated over the course of the disease. These changes associated with autoimmunity may be linked to autoantibody production during babesiosis and malaria.

Besides IgG autoantibodies, Carli et al. [39] detected IgM anti-erythrocyte antibodies in dogs infected with *B. vogeli*. Similarly, IgM antibodies to RBCs have been detected in humans infected with *P. falciparum* [175]. IL-6 is also involved in stimulating the production of IgM antibodies. However, IFN-γ inhibits both IgM and IgG_1_ production, but induces IgG_2a_ and IgG_3_ [188]. As IFN-γ is an essential cytokine during Th1 responses [192], this may partially explain a reason for the detection of mainly IgG autoantibodies in most studies examining anti-erythrocyte antibodies during babesiosis and malaria. Another explanation may be the short half-life of IgM in comparison to various subclasses of IgG antibodies [39,169,170,171,172,173,174,200].

## 6. Antibody-Dependent Cellular Cytotoxicity

Antibody-dependent cellular cytotoxicity (ADCC) is an immunological defense mechanism. Antibodies bound to a target cell mediate the formation of an immunological synapse between a cytotoxic cell and the target cell, and after the synapse is formed cytotoxic factors such as granulysin, granzymes, and perforin are secreted by the cytotoxic cell, which leads to lysis of the target cell [201]. Goff et al. [191] demonstrated that ADCC occurs during bovine babesiosis caused by *B. bovis* and is mediated by IgG_1_. Similar results were observed in human malaria caused by *P. falciparum* [202], with the authors demonstrating that IgG from humans living in regions endemic for malaria induced lysis of parasitized RBCs through NK-mediated ADCC. However, this reaction was highly selective and led only to the destruction of infected erythrocytes. According to Arora et al. [202], NK-driven ADCC leading to the lysis of infected RBCs had not been previously demonstrated, although previous studies had shown that NK cells contribute to the lysis of *Plasmodium*-infected RBCs and erythrocytic stages of the parasite [203,204]. To the best of the authors’ knowledge, there is no study examining the role of ADCC during canine babesiosis. Such work could elucidate if this mechanism participates in the development of anemia or only contributes to the killing of the parasite in infected animals.

NK cells are the main effectors in ADCC. The binding of the Fab region of IgG to an antigen, and of the antibodies’ Fc fragment to the Fcγ receptor on a NK cell, starts the reaction [205]. IL-15 is a cytokine required for development and maintenance of NK cells [206]. Another cytokine, IL-2, induces NK cell proliferation [207]. However, IL-2 also induces regulatory T cells to inhibit NK cell function, whereas IL-15 does not stimulate these regulatory cells and enhances NK cell cytotoxicity. NK cell function is also inhibited by IL-10 [206]. Moreover, as discussed above, the cytokine GM-CS enhances ADCC in other cells, e.g., neutrophils [208].

All four of these cytokines have been examined over the course of canine babesiosis. Higher concentrations of IL-2 were observed only at the beginning of the disease caused by *B. rossi* or *B. canis* [28,30]. No association between the severity of disease and the IL-2 level in infected animals has been reported [79]. The serum concentration of IL-10 was higher in infected dogs, but again no association with the severity of the disease was reported [28,79]. IL-15 was elevated in *B. canis*-infected dogs, but not during *B. rossi* infection [27,30,79], and there was no association between IL-15 and complications of the disease in babesiosis caused by *B. canis* [30]. As mentioned earlier, CSF-2 showed increased levels only at the beginning of *B. canis* and *B. rossi* infections [28,30].

Increased expression of IL-15 mRNA in the spleen has been detected during *B. bovis* infection [209]. Mice genetically deficient in IL-15 showed delayed clearance of *P. chabaudi* during experimental murine malaria [210]. Moreover, as mentioned earlier in this review, CSF-2-deficient mice experimentally infected with *P. chabaudi* had higher peak parasitemia and mortality in comparison to wild-type-infected mice, but both groups had equivalent levels of anemia [83]. The results of those studies together with the results of studies on canine babesiosis [27,28,30,79], and the study on ADCC in *P. falciparum* malaria [202], indicate that during both malaria and babesiosis, IL-15, CSF-2, and cytotoxicity mediated by NK cells and neutrophils contribute to the removal of the parasite, but probably not to the development of anemia.

## 7. Complement System

Over 30 plasma and cell membrane proteins belong to the complement system, which is one of the major mechanisms of innate immunity. Complement can be activated through three major pathways: the classical, lectin, and alternative pathways (positively regulated by properdin). Antibodies bound to an antigen contribute only during the classical pathway of complement activation. Several effector mechanisms are common to all pathways of complement activation, including the formation of the membrane attack complex (C5b-9) leading to perforation of the cell membrane, proteolytic activation of C3, C4, and C5 to generate anaphylotoxins (C4a, C3a, and C5a) that engage granulocytes and monocytes/macrophages to cytokine production, phagocytosis, degranulation, and oxidative burst, and opsonization of the target cell and binding to complement receptors on the surface of phagocytic cells [211]. The complement system may be involved in autoimmunity through stimulation of IL-1β secretion [212]. Increased concentrations of this cytokine have been demonstrated both during bovine and murine babesiosis and human malaria [160,213,214]. Moreover, upregulation of the expression of genes related to erythropoesis, heme, haptoglobin, peroxiredoxin 2 (antioxidant enzyme), arachidonate 15-lipoxygenase, and cytosolic phospholipase 2 syntheses, and related to positive regulation of IL-1β production and IFN-γ signaling, have been observed in *B. rossi*-infected dogs during disease progression [93].

In 1976, Chapman and Ward [165] demonstrated depletion of complement components in rats infected with *B. rodhaini*. The same authors detected that *B. rodhaini* used components of complement (C3 and C5) to invade human RBCs [215]. However, further studies have not confirmed these observations. Seinen et al. [216] showed that complement does not play an essential role in the development of *B. rodhaini* in BALB/c mice. Similar results were obtained by Levy et al. [217,218] in studies examining the complement system and bovine erythrocytes infected with *B. bovis*. Levy et al. [218] also demonstrated the occurrence of both classical and alternative pathways of complement activation during *B. bovis* infection in vitro. To the best of the authors’ knowledge, the influence of complement activation on anemia development has not been studied in canine babesiosis. However, according to Köster et al. [7], immunoglobulin and complement may contribute to development of anemia during canine babesiosis. Kuleš et al. [219] showed increased expression of apolipoprotein A-IV and complement component 3 in sera collected from dogs infected with *B. canis*. Similar results were observed in another proteomic study on serum proteins in mice infected with *B. microti* [220]. In a further proteomic study of canine babesiosis, Kuleš et al. [221] observed differences between dogs infected with *B. canis* that developed multiple organ dysfunction syndrome compared to *B. canis*-infected dogs that developed systemic inflammatory response syndrome. A decrease in complement inhibitors (such as C4-binding protein or complement receptor type 1) leading to prolonged activation of complement was observed in the former cohort. Another study on serum proteins in dogs infected with *B. canis* showed hyper-β_2_-globulinemia, which may result from an increased C3a protein concentration during canine babesiosis [222]. The results of these studies indicate that complement is activated during canine babesiosis. However, its contribution to the development of anemia requires further studies.

An experimental study on human malaria showed increased complement activation in volunteers infected with *P. falciparum* [223]. Behet et al. [224] showed that complement contributed to the antibody-mediated response in volunteers who were immunized with *P. falciparum* sporozoites. In 15 out of 16 volunteers, sporozoite-specific IgM and IgG antibodies were induced, and complement was deposited on sporozoite surfaces. The membrane permeability of sporozoites was found to be increased in the presence of antibodies and activated complement. Moreover, a study by Raballah et al. [225] showed that mutations in the C5 gene are associated with severe anemia in malaria. These results indicate that complement contributes to the control of the infection, with no evidence that it leads to the development of anemia.

Besides the three major complement activation pathways, serine proteases such as kallikrein and thrombin (both involved in coagulation) may also cleave C5 and C3, generating anaphylotoxins such as C5a and C3a [211]. These proteases also contribute to the patho-mechanism of disseminated intravascular coagulation (DIC), which has been recognized in canine babesiosis and human malaria. This coagulopathy has been associated with the severity of disease and higher mortality in both infections [12,226,227,228,229,230]. Thus, complement activation in some of these infections may also be independent of antibodies. Moreover, DIC contributes to the sequestration of erythrocytes in micro-vessels, in this way participating in decreasing the number of RBCs during both malaria and babesiosis [133,227,231].

In sepsis and related diseases, such as severe COVID-19 and malaria, complement system activation is associated with a life-threatening cytokine storm which is caused by an excessive immune response, leading to increased release of cytokines and chemokines such as TNF-α, IFN-γ, IL-1, IL-6, IL-12, IL-18, CCL2, CXCL8, and CXCL10, among others [232,233,234]. As mentioned earlier, increased production of these pro-inflammatory cytokines and chemokines has been observed during babesiosis [27,28,30,136,150,160]. Keshari et al. [235] demonstrated that inhibition of C5 anaphylotoxin blocks the cytokine storm in *Escherichia coli* sepsis in vitro. Moreover, in a study of C5aR-deficient mice (no expression of the gene for C5a receptor), production of IL-1β and CCL2 was decreased, while in another study a C5aR antagonist decreased IL-1β, CCL2, and TNF-α production [236,237]. Thus, increased production of pro-inflammatory cytokines and chemokines during canine babesiosis may result from complement activation. However, according to Jarczak and Nierhaus [232], the chemokine IL-8, as well as the cytokine IL-6, may also be involved in activation of the complement system in the brain by increasing blood–brain barrier permeability, leading to cerebral oedema. This may lead to further pro-inflammatory changes and increased release of pro-inflammatory cytokines and chemokines [232].

Moreover, activation of complement may occur via the alternative pathway [238]. This pathway is always active at a low level. This low level of activation results from the short half-life (about 90 s) of C3 convertases and the action of complement inhibitor factor H, which accelerates the decay of these convertases. Complement inhibitor factor H and other regulatory proteins protect host cells from destruction by complement [238,239]. During an infection, properdin, a plasma protein that positively regulates the complement system, is released in higher amounts by various cells, such as leukocytes, adipocytes, and endothelial cells. Most of these cells constitutively secrete properdin; however, neutrophils are the most important source of properdin. Increased concentration of properdin in the plasma is a result of neutrophil degranulation. The rapid release of properdin from neutrophils may be stimulated by bacterial lipopolysaccharides and by cytokines and chemokines such as TNF-α, CSF-2, CSF-3, IFN-α, and CXCL8 [238]. As presented above, TNF-α, CSF-2, and CXCL8 have been shown to be at increased levels during both malaria and babesiosis. Thus, it is possible that in both infections, these cytokines and chemokines participate in the alternative pathway of complement activation, which could contribute to the intravascular hemolysis observed in both diseases. Further, as antibodies are not engaged in complement alternative pathway activation, this can explain the lack of association between the severity of anemia and autoantibodies in human malaria that was mentioned earlier [186,187]. Although Chapman and Ward [165] observed depletion of complement components in rats infected with *B. rodhaini*, the authors did not detect depletion from the properdin pathway of complement activation. On the other hand, Pawluczkowycz et al. [240] has demonstrated that hematin, a product released from lysed RBCs, promotes the alternative pathway of complement activation in malaria caused by *P. falciparum*. According to Chen et al. [241], properdin is the key player in hemolytic anemia caused by complement dysregulation.

## 8. Splenic Retention of RBCs

**Figure 2:**
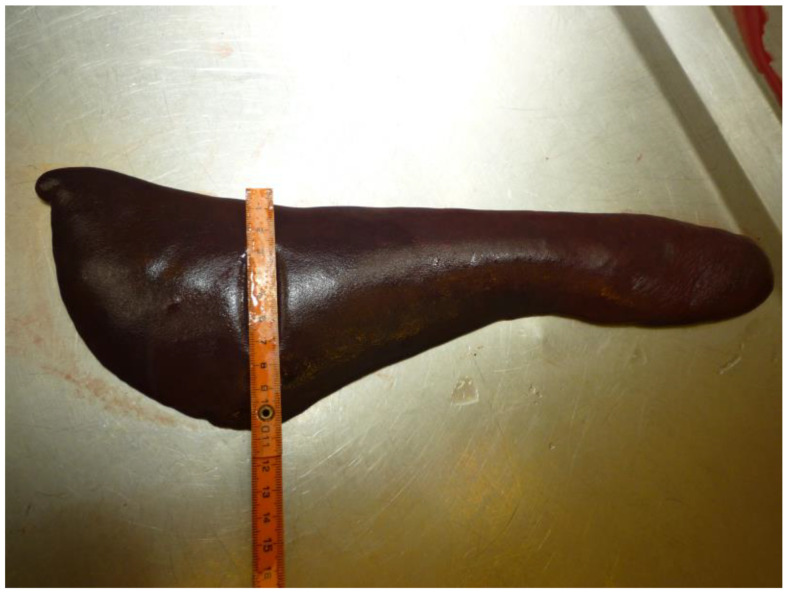
Enlarged spleen from a 4-month-old female German Shepherd which did not survive *B. canis* infection.

Parasitized RBCs are cleared by the spleen in both babesiosis and malaria [8,242,243,244]. Splenomegaly is typical for both diseases in various hosts (Figure 2) [59,72,243,245,246]. In *B. bovis*-infected calves, splenomegaly has been shown to be caused by hyperplasia of both small leukocytes (CD3^+^, CD4^+^, and γδ T cells) and large leukocytes (monocytes, macrophages, dendritic cells, and large NK granular cells). In these animals, hyperplasia was observed in red pulp along with a reduction in white pulp, and a loss in distinction between red pulp and the marginal zone was also observed [246]. Similar results were demonstrated in dogs infected with *B. rossi*, where increased numbers of monocyte-macrophages of bone marrow origin, splenic macrophages, T lymphocytes, mature B lymphocytes, and plasma cells were observed in red pulp, with immature B cells and plasma cells also relocated to red pulp in infected dogs [55].

As in bovine and canine babesiosis, massive hyperplasia of macrophages has been observed in the spleens of mice experimentally infected with *P. chabaudi*. Although the distinction between red and white pulp was lost in traditional hematoxylin-eosin staining, immunohistochemical examination revealed restriction of macrophages to the red pulp in infected animals, while dendritic cells had migrated from red pulp to the areas of white pulp [247].

In human malaria, the spleen is also enlarged. The red pulp is congested with both infected and uninfected RBCs, and there is an increase in the number of macrophages [243]. In rare cases, enlargement of the spleen may lead to rupture of the organ over the course of both human malaria and babesiosis [243,248]. However, according to the authors’ knowledge, rupture of the spleen has not been described in canine babesiosis, except for one case of a dog with asymptomatic babesiosis and an enlarged spleen which ruptured whilst playing with other dogs. Three hours after the incident, the dog had a splenectomy, with babesiosis recognized a few hours after surgery [249].

In human malaria caused by *P. falciparum*, splenic retention of parasitized RBCs results from the fact that these erythrocytes, as less deformable, are not able to pass through very narrow inter-endothelial slits in the slow microcirculation of the splenic red pulp. However, even non-parasitized RBCs are mildly less deformable during an infection [243]. Mourão et al. [176] demonstrated that autoantibodies opsonizing RBCs may decrease the deformability of these cells during *P. vivax* malaria. A similar decrease in deformability has been observed in *P. berghei*- and *B. microti*-infected erythrocytes [250]. Moreover, increased serum ferritin levels, which are observed in human babesiosis, may induce oxidative stress, which can also lead to decreased deformability of RBCs [251,252]. Additionally, it is possible for uninfected RBCs to have pathogen molecules on their surface. Together with infected erythrocytes, due to being less deformable and/or covered with parasite molecules, non-parasitized RBCs are also retained by the spleen and may be phagocytized or lysed by complement [243]. In this way, splenic retention of RBCs may contribute to the development of anemia during both malaria and babesiosis. Moreover, splenomegaly correlates with decreased hematocrit or hemoglobin concentrations in malaria, especially in naïve subjects [243].

In experimental murine malaria, Leisewitz et al. [247] demonstrated increased expression of IFN-γ in splenic dendritic cells at the beginning of infection, which reached a peak on day five. During this early phase of malaria, expressions of TNF-α and IL-10 were static in all splenic cells. A slow increase in IFN-γ expression was observed in macrophages and B lymphocytes in the spleens of infected mice, while increased expression of TNF-α in dendritic cells was observed yet was not significant. Additionally, IL-10 expression did not increase in splenic cells [247]. Additional results in experimental bovine babesiosis were observed by Goff et al. [253]. Increased expression of IL-12 and IFN-γ was observed in splenic aspirates of calves and adult cows infected with *B. bovis*. In young animals, the increase occurred just after infection, while in adult cows it was delayed, being observed five days later [253]. Increased expression of TNF-α in splenic mononuclear cells occurred soon after infection, both in young and adult animals. In young animals, TNF-α peaked at higher levels and started to decrease when IL-10 peak expression was achieved on the ninth day of infection; alternatively, in adults, TNF-α expression started to decrease before IL-10 peaked. Expression of inducible nitric oxide synthase was detected only in calves, and nitric oxide production started earlier and was higher than in adult cows. In both young and adult animals, hematocrit decreased by about 50%; however, only young animals survived the infection. The authors of the study concluded that nitric oxide played a role in controlling the infection during an initial innate Th1 response, but also transiently downregulated the Th1 response. Moreover, IL-12 and IFN-γ production prior to IL-10 production protects the host [253]. Although there are differences between results of studies examining murine malaria and bovine babesiosis, likely resulting from different hosts and pathogens, both works demonstrate the importance of IFN-γ in the splenic response to these infections. Similarly, changes in the spleen architecture associated with destruction of the marginal zone with lysed RBCs and increased plasma concentrations of IFN-γ were observed in *B. microti*-infected mice [59].

In human malaria, hyperreactive malarial splenomegaly has been associated with significantly increased concentrations of IgM, anti-parasite IgG, IL-10, and IFN-γ [254]. According to Del Portillo et al. [255], increased splenic levels of IFN-γ result from the action of IL-12 produced by dendritic cells during the early phase of malaria. IL-12 also causes proliferation of T lymphocytes. Increased production of IFN-γ induces progenitor cells in the bone marrow, which differentiate to monocytes migrating to the spleen during acute *Plasmodium* infection [255]. Moreover, Lacerda-Queiroz et al. [256] demonstrated that increased production of IFN-γ by T cells was associated with apoptosis of red pulp splenic macrophages, dendritic cells, NK cells, and the death of mice experimentally infected with *P. yoelii nigeriensis*. As increased IFN-γ has been observed in experimental canine babesiosis [27], it is possible that IFN-γ leads to similar changes in the spleen of dogs with babesiosis. However, other mechanisms cannot be excluded. Brown et al. [94] did not observe increased IFN-γ or CXCL10 above minimum detectable concentrations in experimental infection with *B. gibsoni*. However, only two dogs were infected in that experiment [94].

## 9. Sequestration of RBCs

Sequestration of erythrocytes in micro-vessels has been observed both in malaria and babesiosis [257,258]. This results from a cyto-adhesive phenomenon, and in babesiosis caused by *B. bovis,* this is partially caused by variant erythrocyte surface antigen 1 (VESA1), while proteins of the *P. falciparum* erythrocyte membrane protein 1 (*Pf*EMP1) family have a role during malaria [258,259]. These proteins act as ligands for receptors on the endothelial cell surface [258,259]. Although other bovine *Babesia* species such as *B. bigemina* and *B. divergens* express VESA proteins (VESA1 or VESA2), Jackson et al. [260] reported that only *B. bovis* infection leads to cytoadherence of infected RBCs and sequestration of erythrocytes in the microvasculature of cattle [260]. However, according to Krause et al. [9], cytoadherence of erythrocytes occurs in infections caused by *B. bigemina*. Moreover, this phenomenon has been reported as present over the course of infections caused by *B. rodhaini* in mice, *B. duncuni* in hamsters, *B. divergens* in humans, and *B. canis* in dogs [9]. Sequestration of infected RBCs has also been observed in canine babesiosis caused by *B. canis* and *B. rossi* [8,72,133], and using electron microscopy, cytoadherence of infected RBCs has been recognized in *B. rossi* infection [71]. In 2017, Eichenberger et al. [261] identified a high number of transcribed VESA genes and their products in the *B. canis*-secreted proteome. According to these authors, cytoadherence and sequestration of *Babesia*-infected erythrocytes in dogs are another explanation for the lack of the association between the severity of infection and the level of parasitemia [261].

Besides parasite proteins on the surface of infected RBCs causing adherence to endothelial cells, *Babesia*-infected erythrocytes can be sequestered in micro-vessels through the action of cell adhesion molecules (CAMs) [262]. In human malaria, direct sequestration of erythrocytes is caused by expression of intercellular adhesion molecule 1 (ICAM-1) on the surface of endothelial cells [263,264]. This molecule also participates in interactions between leukocytes and between leukocytes and endothelial cells. Moreover, ICAM-1 participates in transendothelial migration of leukocytes and adhesion-dependent oxidative burst. Its rapid increased expression is induced by TNF-α, IFN-γ, IL-1α, IL-1β, IL-6, HMGB-1, and reactive oxygen species. Activation of ICAM-1 on endothelial cells leads to increased production of CXCL8 and vascular cell adhesion molecule 1 (VCAM-1), and activation on macrophages leads to increased production of IL-1 [140,264,265]. VCAM-1 is also involved in sequestration of infected RBCs in micro-vessels over the course of malaria. This molecule, like ICAM-1, CD36, and cytokine-activated endothelial protein C, is a receptor that binds *Pf*EMP1 [266,267,268]. VCAM-1 participates in the migration of leukocytes from blood vessels to tissues during infections [269]. Its expression is activated by high levels of reactive oxygen species, oxidized low-density lipoprotein, hyperglycemia, and stimulation of toll-like receptors on endothelial cells. Pro-inflammatory cytokines such as TNF-α, IL-1β, and HMGB-1 activate VCAM-1 by induction of the generation of reactive oxygen species, which stimulates nuclear factor κB. This activation can be blocked by superoxide dismutase, antioxidants (N-acetylocystine or α-tocopherol), and nitric oxide, which interacts with superoxide [140,270].

Cerebral and placental malaria are the best-known complications of *Plasmodium* infections, which are associated with cytoadherence and RBC sequestration in micro-vessels [271,272]. However, autopsies performed in 317 women who did not survive *P. falciparum* malaria demonstrated massive accumulation of infected erythrocytes in most visceral capillaries. Moreover, in cases where placentas were available, the study showed coexistence of cerebral and placental sequestration of infected RBCs [273]. This study showed sequestration of erythrocytes not only in the brain and placenta, but also in other micro-vessels. Similar results showing congestion and sequestration of RBCs in various organs have been observed in canine babesiosis [72,133,274,275]. Moreover, activation of endothelial cells and increased expression of HMGB-1, ICAM-1, and VCAM-1 has been detected in canine babesiosis [29,275]. As mentioned above, increased concentration or expression of pro-inflammatory cytokines TNF-α, IFN-γ, and HMGB-1 occurs during canine babesiosis. Thus, it seems probable that CAMs may contribute to the sequestration of RBCs in micro-vessels during the disease in infected dogs.

DIC and NETosis are other complications which may participate in the sequestration of erythrocytes in microvasculature in malaria, and probably in babesiosis. However, NETosis has not been studied in *Babesia* infections [12,226,227,228,229,230,276]. Activation of neutrophils and release of NETs have been observed in various human parasitic infections, including malaria, leishmaniosis, onchocerciasis (induced by endosymbionts *Wolbachia*), and toxoplasmosis, as well as in bacterial, fungal, and viral infections, in experimental animal models, in in vitro experiments, and in dogs with sepsis [276,277,278,279,280,281,282,283,284]. It seems probable that similar mechanisms to those observed in malaria are involved in sequestration of RBCs in babesiosis. Thus, the development of NETosis in various babesiosis, including canine babesiosis, is also possible.

In NETosis, endothelial contact with pathogens leads to sequestration and activation of leukocytes. Monocytes, activated by the expression of tissue factor, induce fibrin formation. P-selectin, ICAM-1, and CXCL1 bind and activate neutrophils to secrete decondensed chromatin, which is spread as strands of DNA bound to histones, myeloperoxidase, and neutrophil elastase. These strands of DNA and proteins form NETs, in which both pathogens and blood cells are trapped [285,286]. Moreover, interactions between activated platelets and neutrophils also induce the release of NETs [283]. Yago et al. [286] showed that NET formation promotes deep vein thrombosis in mice. Delabranche et al. [287] demonstrated that NETosis was associated with the development of DIC in humans with septic shock. Although NETosis has not been studied in canine babesiosis, it has been recognized in dogs with sepsis. Thus, it seems probable that it may develop together with DIC in infected dogs. Possible NETosis development in this disease leading to neutrophil sequestration, trapping, and consumption, may be another explanation for neutropenia in infected dogs. This suggestion is in agreement with the proposition of Kuleš et al. [29], who speculated that increased expression of ICAM-1 and VCAM-1 were possible causes of neutropenia in canine babesiosis. Moreover, NETosis, in which platelets participate, is also a potential explanation as one of the mechanisms for highly prevalent thrombocytopenia in canine babesiosis, which occurs in almost 100% of infected dogs, whereas DIC has been recognized only in a few studies in about 20% of dogs with babesiosis [12,228,288]. However, a hypocoagulable state has been rarely observed over the course of the disease [8].

Increased fibrinolysis has been detected in canine babesiosis [29,230]. In 2013, Goddard et al. [230] demonstrated an increased D-dimer concentration, a product of the fibrinolytic pathway [289], in dogs infected with *B. rossi*, with the concentration higher in non-survivors. In 2017, Kuleš et al. [29] showed that dogs infected with *B. canis* had an increased concentration of soluble urokinase plasminogen activator receptor (suPAR), an increased plasminogen activator inhibitor-1 concentration, and increased plasminogen activity six days after treatment with imidocarb. Moreover, dogs with complicated babesiosis had a higher concentration of a proenzyme, thrombin activatable fibrinolysis inhibitor, in comparison to dogs with uncomplicated disease [29]. However, it should be emphasized that the receptor for urokinase plasminogen activator is expressed by neutrophils, monocytes, macrophages, and activated T lymphocytes, and increased uPAR expression and serum suPAR concentrations are typical for inflammatory and infectious diseases [290,291]. Nevertheless, the results of the studies of Goddard et al. [230] and Kuleš et al. [29] indicate coagulant disorders, and together with the results of the studies by Máthé et al. [12], Ruiz de Gopegui et al. [228], and Barić Rafaj et al. [229], they indicate the presence of both hypercoagulable and hypocoagulable states in canine babesiosis.

As mentioned above, increased ICAM-1 expression is induced by TNF-α, IFN-γ, IL-1α, IL-1β, IL-6, HMGB-1, and reactive oxygen species, and the result of increased ICAM-1 expression is increased expression of VCAM-1 and production of IL-8 [140,264,265]. However, IFN-γ induces ICAM-1 expression mainly in epithelial cells, and endothelial expression requires protein kinase C action [292,293]. On the other hand, IL-4 and IL-10 decrease the expression of ICAM-1, and IL-10 decreases VCAM-1 expression [294,295]. In an experimental study of IL-10-deficient mice with chronic colitis, these CAMs were highly expressed in comparison to the control group [295]. Anti-inflammatory IL-10 expression is increased during canine babesiosis; however, it seems this increase is insufficient to functionally offset the influence of pro-inflammatory cytokines on CAM induction [79]. Most of the pro-inflammatory cytokines that induce CAMs have been studied during canine babesiosis and show an increased concentration, along with an increased concentration of the chemokine IL-8 [27,29,30,79,89,136,150]. However, in one study on canine babesiosis caused by *B. rossi,* the IL-8 concentration was lower in infected dogs [28]. Atkinson et al. [27] demonstrated in an experimental infection that the CXCL8 concentration was dependent on the parasite dose and changed over the course of the disease. Moreover, Smith et al. [93] showed that the maximal IL-8 concentration was associated with peak parasitemia. The studies of Smith et al. [93] and Atkinson et al. [27] may explain the discrepancies observed between studies on *B. canis* and *B. rossi* infections in which opposite correlations between hematocrit and IL-8 were observed [30,79]. Increased concentrations of pro-inflammatory cytokines such as TNF-α, IFN-γ, IL-6, and HMGB-1 in canine babesiosis, together with the results of the studies of Eichenberger et al. [261] on VESA proteins, and Kuleš et al. [29] on ICAM-1 and VCAM-1 in canine babesiosis, indicate sequestration of infected RBCs over the course of the disease. Moreover, neutropenia and increased IL-8 in *Babesia*-infected dogs may indicate possible development of NETosis [27,30,35,36]. This, together with platelet activation and hypercoagulable states in canine babesiosis, may indicate sequestration of both infected and uninfected erythrocytes in micro-vessels, and it is probable that the chemokine CXCL4 participates in this pathology observed in canine babesiosis [12,29,129,228,229].

Regarding sequestration of RBCs in micro-vessels, it seems probable that both infected and uninfected erythrocytes are trapped in micro-vessels by various mechanisms, including the actions of VESA proteins and CAMs, and possibly with the contribution of NETs and DIC. In this way, the RBC count may decrease in canine babesiosis. However, a further study of the association between anemia and sequestration is required to show the degree of participation of RBC sequestration in the development of anemia in infected dogs. Moreover, sequestration of various blood cells in micro-vessels together with DIC and presumptive NETosis contribute to tissue hypoxia, which leads to other complications over the course of the disease.

## 10. Conclusions

Direct destruction of infected RBCs by the parasite is not the main cause of anemia in canine babesiosis. The lack of association between the levels of parasitemia and anemia in *Babesia*-infected dogs indicates that immune responses contribute to decreasing the RBC count. While low venous parasitemia together with severe anemia may be caused by sequestration of infected RBCs in micro-vessels, the immune response nevertheless leads to both killing of the parasite and erythrocyte destruction. Phagocytosis, oxidative damage of RBCs, and the complement system all participate in the development of anemia. These processes are driven by pro-inflammatory cytokines and chemokines. The contributions of these cytokines and chemokines in the development of anemia during canine babesiosis are presented in Table 1 and Table 2.

Increased phagocytic activity of splenic macrophages and neutrophils, and peripheral granulocytic phagocytosis in small vessels, lead to a decrease in the number of RBCs in infected dogs. Moreover, macrophages and probably platelets contribute to oxidative damage of RBCs over the course of the disease.

Antibodies to phosphatidylserine and disregard of “do not eat me” signals are likely involved in antibody-dependent lysis of RBCs during canine babesiosis. Opsonization of erythrocytes by antibodies may lead to complement activation, antibody-dependent cellular cytotoxicity, erythrophagocytosis, and oxidative damage of RBCs. However, ADCC appears to contribute to the removal of the parasite, but not to the development of anemia. Increased production of pro-inflammatory cytokines and chemokines in *Babesia*-infected dogs may partially result from complement activation.

Both parasitized and non-parasitized RBCs are less deformable over the course of infection. RBCs that are less deformable and/or covered with a parasite, including non-parasitized RBCs, may be retained by the spleen, phagocytized by macrophages and neutrophils, or lysed by complement. Thus, splenic retention may contribute to the development of anemia during babesiosis.

Sequestration of erythrocytes in micro-vessels may also contribute to decreased RBC counts. This process is facilitated by VESA proteins and CAMs, and in some cases by DIC. Additionally, it is possible that NETosis also plays a role in sequestration of RBCs in micro-vessels during canine babesiosis.

All these mechanisms involved in the development of anemia are driven by pro-inflammatory cytokines and chemokines. Moreover, delayed or insufficient production of anti-inflammatory cytokines, causing an imbalance between pro- and anti-inflammatory actions, favors these patho-mechanisms.

While this review has highlighted a number of mechanisms that can contribute to the development of anemia during canine babesiosis (along with the role of specific cytokines and chemokines), there still remains several gaps in the knowledge of how canine babesiosis progresses. Consequently, further studies of this disease examining the involvement of immune responses in the development and promotion of anemia during canine babesiosis, with a focus on the roles of cytokines and chemokines that mediate these responses, will further our understanding of this disease. Moreover, the study of NETosis in canine babesiosis and the association between anemia and sequestration of RBCs in micro-vessels is needed to discern if trapping of blood cells in microvasculature significantly contributes to the development of anemia.

## Figures and Tables

**Table 1:** Possible roles of pro-inflammatory cytokines in the development of anemia in canine babesiosis.

Cytokines	Action	Reference
TNF-α	Induction of CXCL8 and CXCL1 gene expression. Together with IFN-γ, induction of monocyte to macrophage differentiation. Stimulation of nitric oxide synthesis. Increased expression of CSF-2. Contribution to secretion of properdin leading to alternative pathway of complement activation. Increased expression of ICAM-1 and VCAM-1.	[73,74,75,76,81,140,238,264,265,270]
IFN-γ	Contribution to classical activation of macrophages leading to release of IL-6 and TNF-α. Contribution to splenic response to infection (induction of progenitor cells in bone marrow which differentiate to monocytes migrating to the spleen). Increased expression of ICAM-1.	[140,145,255,264,265]
IL-6	Influence on development of oxidative stress. Stimulation of IgG_1_ production. Activation of Th1 lymphocytes. Stimulation of IgM production (inhibited by IFN-γ). Contribution to activation of complement system. Increased expression of ICAM-1.	[140,152,153,154,188,189,192,195,232,264,265]
IL-12	Together with IL-18, triggering IFN-γ and TNF-α action. In synergy with IL-18, inducing production of mitochondrial reactive oxygen species.	[99,102]
IL-18	Together with IL-12, triggering IFN-γ and TNF-α action. In synergy with IL-12, inducing production of mitochondrial reactive oxygen species.	[99,102]
GM-CSF (CSF-2)	Contribution to classical activation of macrophages. Contribution to the recruitment of neutrophils and monocytes to the site of infection, and in mobilization of monocytes from bone marrow. Stimulation of production of MCP-1 by neutrophils and macrophages. Enhanced ADCC by neutrophils. Contribution in secretion of properdin leading to alternative pathway of complement activation.	[81,85,208,238]
HMGB-1	Activation of neutrophil adhesive and migratory functions, and production of reactive oxygen species. Increased expression of ICAM-1 and VCAM-1.	[140,141,264,265,270]
IL-4	Stimulation of production and secretion of IgG_1_ and IgE (IgE production inhibited by IL-8). Increased production of IL-6. Decreased expression of ICAM-1.	[188,190,196,197,294,295]
IL-15	Development of NK cells and induction of their cytotoxicity.	[206]
IL-2	Induction of NK cell proliferation. Induction of regulatory T cells to inhibit NK cell function.	[206,207]
IL-1	Increased expression of ICAM-1 and VCAM-1.	[140,264,265,270]

**Table 2:** Possible roles of chemokines in the development of anemia in canine babesiosis.

Chemokines	Action	Reference
IL-8 (CXCL8)	Induction of chemotaxis of neutrophils, their activation, and stimulation of phagocytosis. Potentiating neutrophil oxidative burst. Contribution to classical activation of complement system. Contribution to secretion of properdin leading to alternative pathway of complement activation.	[92,95,96,139,232,238]
MCP-1 (CCL2)	Contribution to classical activation of macrophages. Chemoattractant for monocytes, NK cells, and neutrophils.	[77,78]
KC (CXCL1)-like	Induction of chemotaxis of neutrophils, their activation, and stimulation of phagocytosis. Together with ICAM-1 and P-selectin, activates neutrophils to form NETs.	[92,95,96,285,286]
PF-4 (CXCL4)	Platelet activation and induction of production of mitochondrial reactive oxygen species.	[130]
IP-10 (CXCL10)	Contribution in the recruitment of granulocytes and macrophages, and phagocytosis.	[108]

## Data Availability

Not applicable.

## References

[1] Irwin P.J. (2009). Canine babesiosis: From molecular taxonomy to control. Parasites Vectors.

[2] Matijatko V., Torti M., Schetters T.P. (2012). Canine babesiosis in Europe: How many diseases?. Trends Parasitol..

[3] Baneth G., Cardoso L., Brilhante-Simões P., Schnittger L. (2019). Establishment of *Babesia vulpes* n. sp. (Apicomplexa: Babesiidae), a piroplasmid species pathogenic for domestic dogs. Parasites Vectors.

[4] Marks Stowe D.A., Birkenheuer A.J., Grindem C.B. (2012). Pathology in practice. Intraerythrocytic infection with organisms con-sistent with a large *Babesia* sp. J. Am. Vet. Med. Assoc..

[5] Birkenheuer A., Neel J., Ruslander D., Levy M., Breitschwerdt E. (2004). Detection and molecular characterization of a novel large *Babesia* species in a dog. Vet. Parasitol..

[6] Barash N.R., Thomas B., Birkenheuer A.J., Breitschwerdt E.B., Lemler E., Qurollo B.A. (2019). Prevalence of *Babesia* spp. and clinical characteristics of *Babesia vulpes* infections in North American dogs. J. Vet. Intern. Med..

[7] Kelly P., Köster L.S., Lobetti R.G. (2015). Canine babesiosis: A perspective on clinical complications, biomarkers, and treatment. Vet. Med. Res. Rep..

[8] Jacobson L.S. (2006). The South African form of severe and complicated canine babesiosis: Clinical advances 1994–2004. Vet. Parasitol..

[9] Krause P.J., Daily J., Telford S.R., Vannier E., Lantos P., Spielman A. (2007). Shared features in the pathobiology of babesiosis and malaria. Trends Parasitol..

[10] Djokic V., Rocha S.C., Parveen N. (2021). Lessons Learned for Pathogenesis, Immunology, and Disease of Erythrocytic Parasites: *Plasmodium* and *Babesia*. Front. Cell. Infect. Microbiol..

[11] Klevtsova E., Sackheim J., Spitzer E., Fries B. (2015). Malaria of the North: Outcomes of Human Babesiosis in Long Island, New York After Treatment Guidelines Revision. Open Forum Infect. Dis..

[12] Máthé Á., Vörös K., Papp L., Reiczigel J. (2006). Clinical manifestations of canine babesiosis in Hungary (63 cases). Acta Vet. Hung..

[13] Jiero S., Pasaribu A.P. (2021). Haematological profile of children with malaria in Sorong, West Papua, Indonesia. Malar. J..

[14] Henry B., Roussel C., Carucci M., Brousse V., Ndour P.A., Buffet P. (2020). The Human Spleen in Malaria: Filter or Shelter?. Trends Parasitol..

[15] Kho S., Barber B.E., Johar E., Andries B., Poespoprodjo J.R., Kenangalem E., Piera K.A., Ehmann A., Price R.N., William T. (2018). Platelets kill circulating parasites of all major *Plasmodium* species in human malaria. Blood.

[16] Kotepui M., Kotepui K.U., Milanez G.D., Masangkay F.R. (2020). Reduction in total leukocytes in malaria patients compared to febrile controls: A systematic review and meta-analysis. PLoS ONE.

[17] Ullah I., Ali M.U., Ali S., Rafiq A., Sattar Z., Hussain S. (2018). Hematological Profile of Patients Having Malaria-positive Peripheral Blood Smears: A Cross-sectional Study at a Diagnostic Research Center in Khyber Pakhtunkhwa, Pakistan. Cureus.

[18] Francischetti I.M.B. (2008). Does Activation of the Blood Coagulation Cascade Play a Role in Malaria Pathogenesis?. Trends Parasitol..

[19] Milner D.A. (2018). Malaria Pathogenesis. Cold Spring Harb. Perspect. Med..

[20] Glaharn S., Punsawad C., Ward S.A., Viriyavejakul P. (2018). Exploring pancreatic pathology in *Plasmodium falciparum* malaria patients. Sci. Rep..

[21] Mishra S.K., Behera P.K., Satpathi S. (2013). Cardiac involvement in malaria: An overlooked important complication. J. Vector Borne Dis..

[22] Clark I.A., Budd A.C., Alleva L.M., Cowden W.B. (2006). Human malarial disease: A consequence of inflammatory cytokine release. Malar. J..

[23] Bhutani A., Kaushik R.M., Kaushik R. (2020). A study on multi-organ dysfunction syndrome in malaria using sequential organ failure assessment score. Trop. Parasitol..

[24] Teparrukkul P., Hantrakun V., Imwong M., Teerawattanasook N., Wongsuvan G., Day N.P., Dondorp A.M., West T.E., Limmathurotsakul D. (2019). Utility of qSOFA and modified SOFA in severe malaria presenting as sepsis. PLoS ONE.

[25] Matijatko V., Kiš I., Torti M., Brkljačić M., Kučer N., Rafaj R.B., Grden D., Živičnjak T., Mrljak V. (2009). Septic shock in canine babesiosis. Vet. Parasitol..

[26] Royo J., Rahabi M., Kamaliddin C., Ezinmegnon S., Olagnier D., Authier H., Massougbodji A., Alao J., Ladipo Y., Deloron P. (2019). Changes in monocyte subsets are associated with clinical outcomes in severe malarial anaemia and cerebral malaria. Sci. Rep..

[27] Atkinson B., Thompson P., Van Zyl E., Goddard A., Rautenbach Y., Schoeman J., Mukorera V., Leisewitz A. (2022). Kinetics of the inflammatory response during experimental *Babesia rossi* infection of beagle dogs. Vet. Parasitol..

[28] Goddard A., Leisewitz A., Kjelgaard-Hansen M., Kristensen A.T., Schoeman J. (2016). Excessive Pro-Inflammatory Serum Cytokine Concentrations in Virulent Canine Babesiosis. PLoS ONE.

[29] Kuleš J., Gotić J., Mrljak V., Barić Rafaj R. (2017). Blood markers of fibrinolysis and endothelial activation in canine babesiosis. BMC Vet. Res..

[30] Galán A., Mayer I., Rafaj R.B., Bendelja K., Sušić V., Cerón J.J., Mrljak V. (2018). MCP-1, KC-like and IL-8 as critical mediators of pathogenesis caused by *Babesia canis*. PLoS ONE.

[31] Reyers F., Leisewitz A.L., Lobetti R.G., Milner R.J., Jacobson L.S., Van Zyl M. (1998). Canine babesiosis in South Africa: More than one disease. Does this serve as a model for falciparum malaria?. Ann. Trop. Med. Parasitol..

[32] Leisewitz A.L., Goddard A., Clift S., Thompson P., de Gier J., Van Engelshoven J.M., Schoeman J. (2019). A clinical and pathological description of 320 cases of naturally acquired *Babesia rossi* infection in dogs. Vet. Parasitol..

[33] Fabisiak M., Sapierzyński R., Kluciński W. (2010). Analysis of haematological abnormalities observed in dogs infected by a large *Babesia*. Bull. Vet. Inst. Pulawy.

[34] Adaszek Ł., Winiarczyk S., Skrzypczak M. (2009). The clinical course of babesiosis in 76 dogs infected with protozoan parasites *Babesia canis canis*. Pol. J. Vet. Sci..

[35] Furlanello T., Fiorio F., Caldin M., Lubas G., Solano-Gallego L. (2005). Clinicopathological findings in naturally occurring cases of babesiosis caused by large form *Babesia* from dogs of northeastern Italy. Vet. Parasitol..

[36] Zygner W., Gójska O., Rapacka G., Jaros D., Wędrychowicz H. (2007). Hematological changes during the course of canine babesiosis caused by large *Babesia* in domestic dogs in Warsaw (Poland). Vet. Parasitol..

[37] Leschnik M., Hooijberg E., Tichy A., Leidinger E., Kirtz G. (2012). In-clinic laboratory diagnosis of canine babesiosis (*Babesia canis canis*) for veterinary practitioners in Central Europe. Tierärztl. Prax. Ausg. K Kleintiere Heimtiere.

[38] Miró G., Checa R., Paparini A., Ortega N., González-Fraga J.L., Gofton A., Bartolomé A., Montoya A., Gálvez R., Mayo P.P. (2015). Theileria annae (syn. *Babesia microti*-like) infection in dogs in NW Spain detected using direct and indirect diagnostic techniques: Clinical report of 75 cases. Parasites Vectors.

[39] Carli E., Tasca S., Trotta M., Furlanello T., Caldin M., Solano-Gallego L. (2009). Detection of erythrocyte binding IgM and IgG by flow cytometry in sick dogs with *Babesia canis canis* or *Babesia canis vogeli* infection. Vet. Parasitol..

[40] Eichenberger R.M., Riond B., Willi B., Hofmann-Lehmann R., Deplazes P. (2016). Prognostic Markers in Acute *Babesia canis* Infec-tions. J. Vet. Intern. Med..

[41] Schofield L., Grau G.E. (2005). Immunological processes in malaria pathogenesis. Nat. Rev. Immunol..

[42] Price R.N., Luxemburger C., Simpson J.A., Hkirjaroen L., Nosten F., White N.J., Chongsuphajaisiddhi T., Kuile F.T. (2001). Factors contributing to anemia after uncomplicated falciparum malaria. Am. J. Trop. Med. Hyg..

[43] Jakeman G.N., Saul A., Hogarth W.L., Collins W.E. (1999). Anaemia of acute malaria infections in non-immune patients primarily results from destruction of uninfected erythrocytes. Parasitology.

[44] Zygner W., Gójska-Zygner O., Długosz E., Wędrychowicz H. (2011). Liver enzyme activity in dogs infected with *Babesia canis*. Bull. Vet. Inst. Pulawy.

[45] Otsuka Y., Yamasaki M., Yamato O., Maede Y. (2002). The Effect of Macrophages on the Erythrocyte Oxidative Damage and the Pathogenesis of Anemia in *Babesia gibsoni*-Infected Dogs with Low Parasitemia. J. Vet. Med Sci..

[46] O’Bryan J., Gokhale A., Hendrickson J.E., Krause P.J. (2020). Parasite burden and red blood cell exchange transfusion for babesiosis. J. Clin. Apher..

[47] Puri A., Bajpai S., Meredith S., Aravind L., Krause P.J., Kumar S. (2021). *Babesia microti*: Pathogen Genomics, Genetic Variability, Immunodominant Antigens, and Pathogenesis. Front. Microbiol..

[48] Agina O., Shaari M., Isa N., Ajat M., Zamri-Saad M., Hamzah H. (2020). Clinical Pathology, Immunopathology and Advanced Vaccine Technology in Bovine Theileriosis: A Review. Pathogens.

[49] Da Silva A.S., França R.T., Costa M.M., Paim C.B., Paim F.C., Dornelles G.L., Soares J.F., Labruna M.B., Mazzanti C.M., Monteiro S.G. (2011). Experimental infection with Rangelia vitalii in dogs: Acute phase, parasitemia, biological cycle, clinical-pathological aspects and treatment. Exp. Parasitol..

[50] França R.T., Da Silva A.S., Loretti A.P., Mazzanti C.M., Lopes S.T. (2014). Canine rangeliosis due to Rangelia vitalii: From first report in Brazil in 1910 to current day—A review. Ticks Tick-Borne Dis..

[51] Murase T., Ueda T., Yamato O., Tajima M., Maede Y. (1996). Oxidative Damage and Enhanced Erythrophagocytosis in Canine Erythrocytes Infected with *Babesia gibsoni*. J. Vet. Med Sci..

[52] Schetters T. (2019). Mechanisms Involved in the Persistence of *Babesia canis* Infection in Dogs. Pathogens.

[53] Uribe-Querol E., Rosales C. (2020). Phagocytosis: Our Current Understanding of a Universal Biological Process. Front. Immunol..

[54] Murase T., Maede Y. (1990). Increased erythrophagocytic activity of macrophages in dogs with *Babesia gibsoni* infection. Jpn. J. Vet. Sci..

[55] Henning A., Clift S.J., Leisewitz A.L. (2020). The pathology of the spleen in lethal canine babesiosis caused by *Babesia rossi*. Parasite Immunol..

[56] Van De Maele I., Savary-Bataille K., Gielen I., Daminet S. (2008). An unusual form of canine babesiosis. Am. Jew. Hist..

[57] Radi Z.A., Styer E.L., Frazier K.S. (2004). Electron Microscopic Study of Canine *Babesia Gibsoni* Infection. J. Vet. Diagn. Investig..

[58] Rebar A.H., Lewis H.B., DeNicola D.B., Halliwell W.H., Boon G.D. (1981). Red Cell Fragmentation in the Dog: An Editorial Review. Vet. Pathol..

[59] Djokic V., Akoolo L., Parveen N. (2018). *Babesia microti* Infection Changes Host Spleen Architecture and Is Cleared by a Th1 Immune Response. Front. Microbiol..

[60] Park H., Hong S.-H., Kim K., Cho S.-H., Lee W.-J., Kim Y., Lee S.-E., Park Y. (2015). Characterizations of individual mouse red blood cells parasitized by *Babesia microti* using 3-D holographic microscopy. Sci. Rep..

[61] Shear H.L., Nussenzweig R.S., Bianco C. (1979). Immune phagocytosis in murine malaria. J. Exp. Med..

[62] Ozarslan N., Robinson J.F., Gaw S.L. (2019). Circulating Monocytes, Tissue Macrophages, and Malaria. J. Trop. Med..

[63] Lee S.W., Lee S.E., Chung B.H., Hwang T.J., Shin H.S. (2008). A Case of *Plasmodium vivax* Malaria Associated with Autoimmune Hemolytic Anemia. Infect. Chemother..

[64] Stockham S.L., Kjemtrup A.M., Conrad P.A., Schmidt D.A., Scott M.A., Robinson T.W., Tyler J.W., Johnson G.C., Carson C.A., Cuddihee P. (2000). Theileriosis in a Missouri Beef Herd Caused by Theileria buffeli: Case Report, Herd Investigation, Ultra-structure, Phylogenetic Analysis, and Experimental Transmission. Vet. Pathol..

[65] Court R., Jackson L., Lee R. (2001). Elevated anti-parasitic activity in peripheral blood monocytes and neutrophils of cattle infected with *Babesia bovis*. Int. J. Parasitol..

[66] Bloch E.M., Kumar S., Krause P.J. (2019). Persistence of *Babesia microti* Infection in Humans. Pathogens.

[67] Dale D.C., Wolff S.M. (1973). Studies of the neutropenia of acute malaria. Blood.

[68] Cooke B.M., Mohandas N., Cowman A.F., Coppel R.L. (2005). Cellular adhesive phenomena in apicomplexan parasites of red blood cells. Vet. Parasitol..

[69] Aitken E.H., Alemu A., Rogerson S.J. (2018). Neutrophils and Malaria. Front. Immunol..

[70] Corduneanu A., Ursache T.D., Taulescu M., Sevastre B., Modrý D., Mihalca A.D. (2020). Detection of DNA of *Babesia canis* in tissues of laboratory rodents following oral inoculation with infected ticks. Parasites Vectors.

[71] Leisewitz A., Turner G., Clift S., Pardini A. (2014). The neuropathology of canine cerebral babesiosis compared to human cerebral malaria. Malar. J..

[72] Zygner W., Rodo A., Gójska-Zygner O., Górski P., Bartosik J., Kotomski G. (2021). Disorders in blood circulation as a probable cause of death in dogs infected with *Babesia canis*. J. Vet. Res..

[73] Salim T., Sershen C.L., May E.E. (2016). Investigating the Role of TNF-α and IFN-γ Activation on the Dynamics of iNOS Gene Expression in LPS Stimulated Macrophages. PLoS ONE.

[74] Goff W.L., O’Rourke K.I., Johnson W.C., Lacy P.A., Davis W.C., Wyatt C.R. (1998). The Role of IL-10 in iNOS and Cytokine mRNA Expression During In Vitro Differentiation of Bovine Mononuclear Phagocytes. J. Interf. Cytokine Res..

[75] Lo H.-M., Lai T.-H., Li C.-H., Wu W.-B. (2014). TNF-α induces CXCL1 chemokine expression and release in human vascular endothelial cells in vitro via two distinct signaling pathways. Acta Pharmacol. Sin..

[76] Mukaida N., Mahe Y., Matsushima K. (1990). Cooperative Interaction of Nuclear Factor-κB- and cis-Regulatory Enhancer Binding Protein-like Factor Binding Elements in Activating the Interleukin-8 Gene by Pro-inflammatory Cytokines. J. Biol. Chem..

[77] Gschwandtner M., Derler R., Midwood K.S. (2019). More Than Just Attractive: How CCL2 Influences Myeloid Cell Behavior Beyond Chemotaxis. Front. Immunol..

[78] Miu J., Mitchell A., Ball H., Hunt N. (2006). Chemokines and Malaria Infection. Curr. Immunol. Rev..

[79] Leisewitz A., Goddard A., De Gier J., Van Engelshoven J., Clift S., Thompson P., Schoeman J.P. (2019). Disease severity and blood cytokine concentrations in dogs with natural *Babesia rossi* infection. Parasite Immunol..

[80] Goncalves R., Zhang X., Cohen H., Debrabant A., Mosser D.M. (2011). Platelet activation attracts a subpopulation of effector monocytes to sites of Leishmania major infection. J. Exp. Med..

[81] Ushach I., Zlotnik A. (2016). Biological role of granulocyte macrophage colony-stimulating factor (GM-CSF) and macrophage colony-stimulating factor (M-CSF) on cells of the myeloid lineage. J. Leukoc. Biol..

[82] Yamada-Tanaka M.S., Ferreira-Da-Cruz M.F., Alecrim M.G., Mascarenhas L.A., Daniel-Ribeiro C.T. (1995). Tumor necrosis factor alpha interferon gamma and macrophage stimulating factor in relation to the Severity of *Plasmodium falciparum* malaria in the Brazilian Amazon. Trop. Geogr. Med..

[83] Riopel J., Tam M., Mohan K., Marino M.W., Stevenson M.M. (2001). Granulocyte-Macrophage Colony-Stimulating Factor-Deficient Mice Have Impaired Resistance to Blood-Stage Malaria. Infect. Immun..

[84] Skorokhod O., Barrera V., Mandili G., Costanza F., Valente E., Ulliers D., Schwarzer E. (2021). Malaria Pigment Hemozoin Impairs GM-CSF Receptor Expression and Function by 4-Hydroxynonenal. Antioxidants.

[85] Khajah M., Millen B., Cara D.C., Waterhouse C., McCafferty D.-M. (2011). Granulocyte-macrophage colony-stimulating factor (GM-CSF): A chemoattractive agent for murine leukocytes in vivo. J. Leukoc. Biol..

[86] Goff W.L., Johnson W.C., Parish S.M., Barrington G.M., Elsasser T.H., Davis W.C., Valdez R.A. (2002). IL-4 and IL-10 inhibition of IFN-γ- and TNF-α-dependent nitric oxide production from bovine mononuclear phagocytes exposed to *Babesia bovis* mero-zoites. Vet. Immunol. Immunopathol..

[87] Orecchioni M., Ghosheh Y., Pramod A.B., Ley K. (2019). Macrophage Polarization: Different Gene Signatures in M1(LPS+) vs. Classically and M2(LPS–) vs. Alternatively Activated Macrophages. Front. Immunol..

[88] Couper K.N., Blount D.G., Riley E.M. (2008). IL-10: The master regulator of immunity to infection. J. Immunol..

[89] Mayer I., Bendelja K., Brkljačić M., Crnogaj M., Šmit I., Torti M., Sušić V., Barić Rafaj R., Mrljak V. (2015). Serum levels of the chemokines keratinocyte chemoattractant and interleukin-8 in dogs naturally infected with *Babesia canis canis*. Vet. Arh..

[90] Ioannidis L.J., Nie C.Q., Hansen D.S. (2013). The role of chemokines in severe malaria: More than meets the eye. Parasitology.

[91] Doganyigit Z., Eroglu E., Akyuz E. (2022). Inflammatory mediators of cytokines and chemokines in sepsis: From bench to bedside. Hum. Exp. Toxicol..

[92] Hol J., Wilhelmsen L., Haraldsen G. (2009). The murine IL-8 homologues KC, MIP-2, and LIX are found in endothelial cytoplasmic granules but not in Weibel-Palade bodies. J. Leukoc. Biol..

[93] Smith R.L., Goddard A., Boddapati A., Brooks S., Schoeman J.P., Lack J., Leisewitz A., Ackerman H. (2021). Experimental *Babesia rossi* infection induces hemolytic, metabolic, and viral response pathways in the canine host. BMC Genom..

[94] Brown A., Shiel R., Irwin P. (2015). Clinical, haematological, cytokine and acute phase protein changes during experimental *Babesia gibsoni* infection of beagle puppies. Exp. Parasitol..

[95] Beste M.T., Lomakina E.B., Hammer D.A., Waugh R.E. (2015). Immobilized IL-8 Triggers Phagocytosis and Dynamic Changes in Membrane Microtopology in Human Neutrophils. Ann. Biomed. Eng..

[96] Silva M.T. (2009). When two is better than one: Macrophages and neutrophils work in concert in innate immunity as complementary and cooperative partners of a myeloid phagocyte system. J. Leukoc. Biol..

[97] Babatunde K.A., Adenuga O.F. (2022). Neutrophils in malaria: A double-edged sword role. Front. Immunol..

[98] Burgmann H., Hollenstein U., Wenisch C., Thalhammer F., Looareesuwan S., Graninger W. (1995). Serum Concentrations of MIP-1 α and Interleukin-8 in Patients Suffering from Acute *Plasmodium falciparum* Malaria. Clin. Immunol. Immunopathol..

[99] Yasuda K., Nakanishi K., Tsutsui H. (2019). Interleukin-18 in Health and Disease. Int. J. Mol. Sci..

[100] Kojima S., Nagamine Y., Hayano M., Looareesuwan S., Nakanishi K. (2003). A potential role of interleukin 18 in severe falciparum malaria. Acta Trop..

[101] Mazodier K., Marin V., Novick D., Farnarier C., Robitail S., Schleinitz N., Veit V., Paul P., Rubinstein M., Dinarello C.A. (2005). Severe imbalance of IL-18/IL-18BP in patients with secondary hemophagocytic syndrome. Blood.

[102] Rackov G., Tavakoli Zaniani P., Colomo Del Pino S., Shokri R., Monserrat J., Alvarez-Mon M., Martinez-A C., Balomenos D. (2022). Mitochondrial reactive oxygen is critical for IL-12/IL-18-induced IFN-γ production by CD4+ T cells and is regulated by Fas/FasL signaling. Cell Death Dis..

[103] Suarez C.E., Alzan H.F., Silva M.G., Rathinasamy V., Poole W.A., Cooke B.M. (2019). Unravelling the cellular and molecular pathogenesis of bovine babesiosis: Is the sky the limit?. Int. J. Parasitol..

[104] Ioannidis L.J., Nie C.Q., Ly A., Ryg-Cornejo V., Chiu C.Y., Hansen D.S. (2015). Monocyte- and Neutrophil-Derived CXCL10 Impairs Efficient Control of Blood-Stage Malaria Infection and Promotes Severe Disease. J. Immunol..

[105] Liu M., Amodu A.S., Pitts S., Patrickson J., Hibbert J., Battle M., Ofori-Acquah S., Stiles J.K. (2012). Heme Mediated STAT3 Activation in Severe Malaria. PLoS ONE.

[106] Decker M.-L., Gotta V., Wellmann S., Ritz N. (2017). Cytokine profiling in healthy children shows association of age with cytokine concentrations. Sci. Rep..

[107] Hayney M.S., Henriquez K.M., Barnet J.H., Ewers T., Champion H.M., Flannery S., Barrett B. (2017). Serum IFN-γ-induced protein 10 (IP-10) as a biomarker for severity of acute respiratory infection in healthy adults. J. Clin. Virol..

[108] Cuenca A.G., Wynn J.L., Kelly-Scumpia K.M., Scumpia P.O., Vila L., Delano M.J., Mathews C.E., Wallet S.M., Reeves W.H., Behrns K.E. (2011). Critical Role for CXC Ligand 10/CXC Receptor 3 Signaling in the Murine Neonatal Response to Sepsis. Infect. Immun..

[109] Branch D.R., Leger R.M., Sakac D., Yi Q., Duong T., Yeung R.S.M., Binnington B., Bloch E.M. (2022). Chemokines IP-10/CXCL10 and IL-8/CXCL8 are potential novel biomarkers of warm autoimmune hemolytic anemia. Blood Adv..

[110] Woolley A.E., Montgomery M.W., Savage W.J., Achebe M.O., Dunford K., Villeda S., Maguire J.H., Marty F.M. (2017). Post-Babesiosis Warm Autoimmune Hemolytic Anemia. N. Engl. J. Med..

[111] Herb M., Schramm M. (2021). Functions of ROS in Macrophages and Antimicrobial Immunity. Antioxidants.

[112] Otsuka Y., Yamasaki M., Yamato O., Maede Y. (2001). Increased Generation of Superoxide in Erythrocytes infected with *Babesia gibsoni*. J. Vet. Med Sci..

[113] Morita T., Saeki H., Imai S., Ishii T. (1996). Erythrocyte oxidation in artificial *Babesia gibsoni* infection. Vet. Parasitol..

[114] Crnogaj M., Petlevski R., Mrljak V., Kiš I., Torti M., Küçer N., Matijatko V., Sacer I., Štokoviç I. (2010). Malondialdehyde levels in serum of dogs infected with *Babesia canis*. Vet. Med..

[115] Jacobson L.S., Lobetti R.G., Becker P., Reyers F., Vaughan-Scott T. (2001). Nitric oxide metabolites in naturally occurring canine babesiosis. Vet. Parasitol..

[116] Harvey J.W., Rackear D. (1985). Experimental Onion-Induced Hemolytic Anemia in Dogs. Vet. Pathol..

[117] Chaudhuri S., Varshney J., Patra R. (2008). Erythrocytic antioxidant defense, lipid peroxides level and blood iron, zinc and copper concentrations in dogs naturally infected with *Babesia gibsoni*. Res. Vet. Sci..

[118] Teodorowski O., Winiarczyk S., Tarhan D., Dokuzeylül B., Ercan A.M., Or M.E., Staniec M., Adaszek Ł. (2021). Antioxidant status, and blood zinc and copper concentrations in dogs with uncomplicated babesiosis due to *Babesia canis* infections. J. Vet. Res..

[119] Crnogaj M., Cerón J.J., Šmit I., Kiš I., Gotić J., Brkljačić M., Matijatko V., Rubio C.P., Kučer N., Mrljak V. (2017). Relation of antioxidant status at admission and disease severity and outcome in dogs naturally infected with *Babesia canis canis*. BMC Vet. Res..

[120] Deger S., Deger Y., Bicek K., Ozdal N., Gul A. (2009). Status of Lipid Peroxidation, Antioxidants, and Oxidation Products of Nitric Oxide in Equine Babesiosis: Status of Antioxidant and Oxidant in Equine Babesiosis. J. Equine Vet. Sci..

[121] Esmaeilnejad B., Tavassoli M., Asri-Rezaei S., Dalir-Naghadeh B., Malekinejad H. (2012). Status of lipid peroxidation and anti-oxidant enzymes in goats naturally infected with *Babesia ovis*. Acta Parasitol..

[122] Esmaeilnejad B., Tavassoli M., Asri-Rezaei S., Dalir-Naghadeh B. (2011). Evaluation of antioxidant status and oxidative stress in sheep naturally infected with *Babesia ovis*. Vet. Parasitol..

[123] Saleh M.A. (2009). Erythrocytic oxidative damage in crossbred cattle naturally infected with *Babesia bigemina*. Res. Vet. Sci..

[124] Saleh M.A., Mahran O.M., Al-Salahy M.B. (2011). Corpuscular oxidation in newborn crossbred calves naturally infected with Theileria annulata. Vet. Parasitol..

[125] Erel O., Kocyigit A., Avci S., Aktepe N., Bulut V. (1997). Oxidative Stress and Antioxidative Status of Plasma and Erythrocytes in Patients with Vivax Malaria. Clin. Biochem..

[126] Kulkarni A.G., Suryakar A.N., Sardeshmukh A.S., Rathi D.B. (2003). Studies on biochemical changes with special reference to oxidant and antioxidants in malaria patients. Indian J. Clin. Biochem..

[127] Bilgin R., Yalcin M.S., Yucebilgic G., Koltas I.S., Yazar S. (2012). Oxidative Stress in Vivax Malaria. Korean J. Parasitol..

[128] Goddard A., Leisewitz A.L., Kristensen A.T., Schoeman J.P. (2015). Platelet activation and platelet–leukocyte interaction in dogs naturally infected with *Babesia rossi*. Vet. J..

[129] Goddard A., Leisewitz A.L., Kristensen A.T., Schoeman J.P. (2015). Platelet indices in dogs with *Babesia rossi* infection. Vet. Clin. Pathol..

[130] Annarapu G.K., Nolfi-Donegan D., Reynolds M., Wang Y., Kohut L., Zuckerbraun B., Shiva S. (2021). Heme stimulates platelet mitochondrial oxidant production to induce targeted granule secretion. Redox Biol..

[131] Wilson N.O., Jain V., Roberts C.E., Lucchi N., Joel P.K., Singh M.P., Nagpal A.C., Dash A.P., Udhayakumar V., Singh N. (2011). CXCL4 and CXCL10 predict risk of fatal cerebral malaria. Dis. Markers.

[132] Ghazanfari N., Mueller S., Heath W.R. (2018). Cerebral Malaria in Mouse and Man. Front. Immunol..

[133] Daste T., Lucas M.-N., Aumann M. (2013). Cerebral babesiosis and acute respiratory distress syndrome in a dog. J. Vet. Emerg. Crit. Care.

[134] Nevils M.A., Figueroa J.V., Turk J.R., Canto G.J., Le V., Ellersieck M.R., Carson C.A. (2000). Cloned lines of *Babesia bovis* differ in their ability to induce cerebral babesiosis in cattle. Parasitol. Res..

[135] Cox D., McConkey S. (2009). The role of platelets in the pathogenesis of cerebral malaria. Cell. Mol. Life Sci..

[136] Zygner W., Gójska-Zygner O., Bąska P., Długosz E. (2014). Increased concentration of serum TNF alpha and its correlations with arterial blood pressure and indices of renal damage in dogs infected with *Babesia canis*. Parasitol. Res..

[137] Kontaş T., Salmanoğlu B. (2006). Tumour necrosis factor-α, adenosine deaminase and nitric oxide levels in cattle babesiosis before and after treatment. Bull. Vet. Inst. Pulawy.

[138] Shaio M.F., Lin P.R. (1998). A case study of cytokine profiles in acute human babesiosis. Am. J. Trop. Med. Hyg..

[139] Guichard C., Pedruzzi E., Dewas C., Fay M., Pouzet C., Bens M., Vandewalle A., Ogier-Denis E., Gougerot-Pocidalo M.-A., Elbim C. (2005). Interleukin-8-induced Priming of Neutrophil Oxidative Burst Requires Sequential Recruitment of NADPH Oxidase Components into Lipid Rafts. J. Biol. Chem..

[140] Klune J.R., Dhupar R., Cardinal J., Billiar T.R., Tsung A. (2008). HMGB1: Endogenous Danger Signaling. Mol. Med..

[141] Abu El-Asrar A.M., Alam K., Garcia-Ramirez M., Ahmad A., Siddiquei M.M., Mohammad G., Mousa A., De Hertogh G., Opdenakker G., Simó R. (2017). Association of HMGB1 with oxidative stress markers and regulators in PDR. Mol. Vis..

[142] Paclet M.-H., Laurans S., Dupré-Crochet S. (2022). Regulation of Neutrophil NADPH Oxidase, NOX2: A Crucial Effector in Neutrophil Phenotype and Function. Front. Cell Dev. Biol..

[143] Bartesaghi S., Radi R. (2017). Fundamentals on the biochemistry of peroxynitrite and protein tyrosine nitration. Redox Biol..

[144] Pacher P., Beckman J.S., Liaudet L. (2007). Nitric Oxide and Peroxynitrite in Health and Disease. Physiol. Rev..

[145] Ivanova E.A., Orekhov A.N. (2016). Monocyte Activation in Immunopathology: Cellular Test for Development of Diagnostics and Therapy. J. Immunol. Res..

[146] Perkins D.J., Were T., Davenport G.C., Kempaiah P., Hittner J.B., Ong’Echa J.M. (2011). Severe Malarial Anemia: Innate Immunity and Pathogenesis. Int. J. Biol. Sci..

[147] Day N.P.J., Hien T.T., Schollaardt T., Loc P.P., Van Chuong L., Chau T.T.H., Mai N.T.H., Phu N.H., Sinh D.X., White N.J. (1999). The Prognostic and Pathophysiologic Role of Pro- and Antiinflammatory Cytokines in Severe Malaria. J. Infect. Dis..

[148] Achur R., Punnath K., Dayanand K.K., Chandrashekhar V.N., Kakkilaya S.B., Ghosh S.K., Kumari S.N., Gowda C. (2019). Association between inflammatory cytokine levels and anemia during *Plasmodium falciparum* and *Plasmodium vivax* infections in Mangaluru: A Southwestern Coastal Region of India. Trop. Parasitol..

[149] Awandare G.A., Goka B., Boeuf P., Tetteh J.K.A., Kurtzhals J.A.L., Behr C., Akanmori B.D. (2006). Increased Levels of Inflammatory Mediators in Children with Severe *Plasmodium falciparum* Malaria with Respiratory Distress. J. Infect. Dis..

[150] Zygner W., Gójska-Zygner O., Bąska P., Długosz E. (2015). Low T3 syndrome in canine babesiosis associated with increased serum IL-6 concentration and azotaemia. Vet. Parasitol..

[151] Wilairatana P., Mala W., Milanez G.D.J., Masangkay F.R., Kotepui K.U., Kotepui M. (2022). Increased interleukin-6 levels associated with malaria infection and disease severity: A systematic review and meta-analysis. Sci. Rep..

[152] Petrushevska M., Zendelovska D., Atanasovska E., Eftimov A., Spasovska K. (2021). Presentation of cytokine profile in relation to oxidative stress parameters in patients with severe COVID-19: A case-control pilot study. F1000Research.

[153] Mathy-Hartert M., Hogge L., Sanchez C., Deby-Dupont G., Crielaard J., Henrotin Y. (2008). Interleukin-1β and interleukin-6 disturb the antioxidant enzyme system in bovine chondrocytes: A possible explanation for oxidative stress generation. Osteoarthr. Cartil..

[154] Wassmann S., Stumpf M., Strehlow K., Schmid A., Schieffer B., Böhm M., Nickenig G. (2004). Interleukin-6 Induces Oxidative Stress and Endothelial Dysfunction by Overexpression of the Angiotensin II Type 1 Receptor. Circ. Res..

[155] Peng Y., Yang Q., Gao S., Liu Z., Kong W., Bian X., Li Z., Ye J. (2022). IL-6 protects cardiomyocytes from oxidative stress at the early stage of LPS-induced sepsis. Biochem. Biophys. Res. Commun..

[156] Brown C.O., Salem K., Wagner B.A., Bera S., Singh N., Tiwari A., Choudhury A., Buettner G.R., Goel A. (2012). Interleukin-6 counteracts therapy-induced cellular oxidative stress in multiple myeloma by up-regulating manganese superoxide dismutase. Biochem. J..

[157] Matsuoka Y., Nakayama H., Yoshida R., Hirosue A., Nagata M., Tanaka T., Kawahara K., Sakata J., Arita H., Nakashima H. (2016). IL-6 controls resistance to radiation by suppressing oxidative stress via the Nrf2-antioxidant pathway in oral squamous cell carcinoma. Br. J. Cancer.

[158] Naik E., Dixit V.M. (2011). Mitochondrial reactive oxygen species drive proinflammatory cytokine production. J. Exp. Med..

[159] Bulua A.C., Simon A., Maddipati R., Pelletier M., Park H., Kim K.-Y., Sack M.N., Kastner D.L., Siegel R.M. (2011). Mitochondrial reactive oxygen species promote production of proinflammatory cytokines and are elevated in TNFR1-associated periodic syndrome (TRAPS). J. Exp. Med..

[160] Shoda L.K., Palmer G.H., Florin-Christensen J., Florin-Christensen M., Godson D.L., Brown W.C. (2000). *Babesia bovis*-Stimulated Macrophages Express Interleukin-1β, Interleukin-12, Tumor Necrosis Factor Alpha, and Nitric Oxide and Inhibit Parasite Replication In Vitro. Infect. Immun..

[161] Johnson W.C., Cluff C.W., Goff W.L., Wyatt C.R. (1996). Reactive Oxygen and Nitrogen Intermediates and Products from Polyamine Degradation Are *Babesiacidal* In Vitro. Ann. N. Y. Acad. Sci..

[162] Bogoch I.I., Davis B.T., Hooper D.C. (2012). Severe Babesiosis in a Patient Treated with a Tumor Necrosis Factor α Antagonist. Clin. Infect. Dis..

[163] Taiwo B., Lee C., Venkat D., Tambar S., Sutton S.H. (2007). Can Tumor Necrosis Factor α Blockade Predispose to Severe Babesiosis?. Arthritis Rheum..

[164] Aguilar-Delfin I., Wettstein P.J., Persing D.H. (2003). Resistance to Acute Babesiosis Is Associated with Interleukin-12- and Gamma Interferon-Mediated Responses and Requires Macrophages and Natural Killer Cells. Infect. Immun..

[165] Chapman W.E., Ward P.A. (1976). The Complement Profile in Babesiosis. J. Immunol..

[166] Goff W., Wagner G., Craig T. (1984). Increased activity of bovine ADCC effector cells during acute *Babesia bovis* infection. Vet. Parasitol..

[167] Jacobson R.H., Parrodi F., Wright I.G., Fitzgerald C.J., Dobson C. (1993). *Babesia bovis*: In vitro phagocytosis promoted by immune serum and by antibodies produced against protective antigens. Parasitol. Res..

[168] Flegel W.A. (2015). Pathogenesis and mechanisms of antibody-mediated hemolysis. Transfusion.

[169] Adachi K., Yoshimoto A., Hasegawa T., Shimizu T., Goto Y., Makimura S. (1992). Anti-Erythrocyte Membrane Antibodies Detected in Sera of Dogs Naturally Infected with *Babesia gibsoni*. J. Vet. Med. Sci..

[170] Adachi K., Tateishi M., Horii Y., Nagatomo H., Shimizu T., Makimura S. (1994). Elevated Erythrocyte-Bound IgG Value in Dogs with Clinical *Babesia gibsoni* Infection. J. Vet. Med Sci..

[171] Kanbara T., Azuma M., Hasegawa K., Matsuda H. (1988). Anemia and anti-erythrocyte antibodies developed after repeated injections of sonicated preparations of *Plasmodium berghei* and *Babesia rodhaini*. Zent. Fur Bakteriol. Mikrobiol. Hygiene. Ser. A Med. Microbiol. Infect. Dis. Virol. Parasitol..

[172] Chiou S.-P., Kitoh K., Igarashi I., Takashima Y. (2014). Generation of Monoclonal Autoantibodies from *Babesia rodhaini*-Infected Mice. J. Vet. Med Sci..

[173] Orinda G., Commins M., Waltisbuhl D., Goodger B., Wright I. (1994). A study of autoantibodies to phosphatidyl-serine in *Babesia bovis* and *Babesia bigemina* infections in cattle. Vet. Immunol. Immunopathol..

[174] Góes T., Góes V., Ribeiro M., Gontijo C. (2007). Bovine babesiosis: Anti-erythrocyte antibodies purification from the sera of naturally infected cattle. Vet. Immunol. Immunopathol..

[175] Rosenberg E.B., Strickland G.T., Yang S.-L., Whalen G.E. (1973). IgM antibodies to red cells and autoimmune anemia in patients with malaria. Am. J. Trop. Med. Hyg..

[176] Mourão L.C., Roma P.M., Sultane Aboobacar Jda S., Medeiros C.M., de Almeida Z.B., Fontes C.J., Agero U., de Mesquita O.N., Bemquerer M.P., Braga É.M. (2016). Anti-erythrocyte antibodies may contribute to anaemia in *Plasmodium vivax* malaria by decreasing red blood cell deformability and increasing erythrophagocytosis. Malar. J..

[177] Narurkar R., Mamorska-Dyga A., Nelson J.C., Liu D. (2017). Autoimmune hemolytic anemia associated with babesiosis. Biomark. Res..

[178] Adachi K., Tateishi M., Horii Y., Nagatomo H., Shimizu T., Makimura S. (1995). Immunologic Characteristics of Anti-Erythrocyte Membrane Antibody Produced in Dogs during *Babesia gibsoni* Infection. J. Vet. Med. Sci..

[179] Diaz C., Schroit A. (1996). Role of Translocases in the Generation of Phosphatidylserine Asymmetry. J. Membr. Biol..

[180] Kuypers F.A., De Jong K. (2004). The role of phosphatidylserine in recognition and removal of erythrocytes. Cell. Mol. Biol..

[181] Bernhardt I., Nguyen D.B., Wesseling M.C., Kaestner L. (2020). Intracellular Ca2+ Concentration and Phosphatidylserine Exposure in Healthy Human Erythrocytes in Dependence on in vivo Cell Age. Front. Physiol..

[182] Daniel-Ribeiro C., Deslandes D.C., Ferreira-Da-Cruz M.d.F. (1991). Cross-reactions between idiotypes, *Plasmodium falciparum* derived peptides, dinitrophenyl and beta(2→6) polyfructosan. J. Clin. Lab. Immunol..

[183] Taboada J., Lobetti R., Greene C.E. (2006). Babesiosis. Infectious Diseases of the Dog and Cat.

[184] Rivera-Correa J., Rodriguez A. (2019). Autoimmune Anemia in Malaria. Trends Parasitol..

[185] Fernandez-Arias C., Rivera-Correa J., Gallego-Delgado J., Rudlaff R., Fernandez C., Roussel C., Götz A., Gonzalez S., Mohanty A., Mohanty S. (2016). Anti-Self Phosphatidylserine Antibodies Recognize Uninfected Erythrocytes Promoting Malarial Anemia. Cell Host Microbe.

[186] Mourão L.C., Cardoso-Oliveira G.P., Braga É.M. (2020). Autoantibodies and Malaria: Where We Stand? Insights Into Pathogenesis and Protection. Front. Cell. Infect. Microbiol..

[187] Ventura A.M.R.D.S., Fernandes A.A.M., Zanini G.M., Pratt-Riccio L.R., Sequeira C.G., do Monte C.R.S., Martins-Filho A.J., Machado R.L.D., Libonati R.M.F., de Souza J.M. (2018). Clinical and immunological profiles of anaemia in children and adolescents with *Plasmodium vivax* malaria in the Pará state, Brazilian Amazon. Acta Trop..

[188] Vazquez M.I., Catalan-Dibene J., Zlotnik A. (2015). B cells responses and cytokine production are regulated by their immune microenvironment. Cytokine.

[189] Pioli P. (2019). Plasma Cells, the Next Generation: Beyond Antibody Secretion. Front. Immunol..

[190] Kimata H., Yoshida A., Ishioka C., Lindley I., Mikawa H. (1992). Interleukin 8 (IL-8) selectively inhibits immunoglobulin E production induced by IL-4 in human B cells. J. Exp. Med..

[191] Goff W., Wagner G., Craig T., Long R. (1984). The role of specific immunoglobulins in antibody-dependent cell-mediated cytotoxicity assays during *Babesia bovis* infection. Vet. Parasitol..

[192] Zhu J., Paul W.E. (2008). CD4 T cells: Fates, functions, and faults. Blood.

[193] Xue X., Ren S., Yang X., Masoudi A., Hu Y., Wang X., Li H., Zhang X., Wang M., Wang H. (2021). Protein regulation strategies of the mouse spleen in response to *Babesia microti* infection. Parasites Vectors.

[194] Kotepui K.U., Thirarattanasunthon P., Rattaprasert P., Kotepui M. (2022). A systematic review and meta-analysis of blood inter-leukin-4 levels concerning malaria infection and severity. Malar. J..

[195] Kawano Y., Noma T., Kou K., Yoshizawa I., Yata J. (1995). Regulation of human IgG subclass production by cytokines: Human IgG subclass production enhanced differentially by interleukin-6. Immunology.

[196] Derocq J.-M., Segui M., Poinot-Chaze C., Minty A., Caput D., Ferrara P., Casellas P. (1994). Interleukin-13 stimulates interleukin-6 production by human keratinocytes. FEBS Lett..

[197] Nasu K., Sugano T., Fujisawa K., Arima K., Narahara H., Miyakawa I. (2001). Effects of interleukin-4 on the in-vitro production of cytokines by human endometrial stromal cells. Mol. Hum. Reprod..

[198] Malaguarnera L., Imbesi R.M., Pignatelli S., Simporè J., Malaguarnera M., Musumeci S. (2002). Increased levels of interleukin-12 in *Plasmodium falciparum* malaria: Correlation with the severity of disease. Parasite Immunol..

[199] Otterdal K., Berg A., Michelsen A.E., Yndestad A., Patel S., Gregersen I., Halvorsen B., Ueland T., Langeland N., Aukrust P. (2021). IL-18 and IL-18 binding protein are related to disease severity and parasitemia during falciparum malaria. BMC Infect. Dis..

[200] Vieira P., Rajewsky K. (1988). The half-lives of serum immunoglobulins in adult mice. Eur. J. Immunol..

[201] Prager I., Watzl C. (2019). Mechanisms of natural killer cell-mediated cellular cytotoxicity. J. Leukoc. Biol..

[202] Arora G., Hart G.T., Manzella-Lapeira J., Doritchamou J.Y., Narum D.L., Thomas L.M., Brzostowski J., Rajagopalan S., Doumbo O.K., Traore B. (2018). NK cells inhibit *Plasmodium falciparum* growth in red blood cells via antibody-dependent cellular cytotoxicity. Elife.

[203] Mavoungou E., Luty A., Kremsner P.G. (2003). Natural killer (NK) cell-mediated cytolysis of *Plasmodium falciparum*-infected human red blood cells in vitro. Eur. Cytokine Netw..

[204] Orago A.S.S., Facer C.A. (1991). Cytotoxicity of human natural killer (NK) cell subsets for *Plasmodium falciparum* erythrocytic schizonts: Stimulation by cytokines and inhibition by neomycin. Clin. Exp. Immunol..

[205] Nigro C.L., Macagno M., Sangiolo D., Bertolaccini L., Aglietta M., Merlano M.C. (2019). NK-mediated antibody-dependent cell-mediated cytotoxicity in solid tumors: Biological evidence and clinical perspectives. Ann. Transl. Med..

[206] Rezvani K., Rouce R.H. (2015). The Application of Natural Killer Cell Immunotherapy for the Treatment of Cancer. Front. Immunol..

[207] Liao W., Lin J.-X., Leonard W.J. (2013). Interleukin-2 at the Crossroads of Effector Responses, Tolerance, and Immunotherapy. Immunity.

[208] Martinez Sanz P., van Rees D.J., van Zogchel L.M.J., Klein B., Bouti P., Olsman H., Schornagel K., Kok I., Sunak A., Leeuwenburg K. (2021). G-CSF as a suitable alternative to GM-CSF to boost dinutuxi-mab-mediated neutrophil cytotoxicity in neuroblastoma treatment. J. Immunother. Cancer.

[209] Goff W.L., Johnson W.C., Horn R.H., Barrington G.M., Knowles D.P. (2003). The innate immune response in calves to Boophilus microplus tick transmitted *Babesia bovis* involves type-1 cytokine induction and NK-like cells in the spleen. Parasite Immunol..

[210] Ing R., Gros P., Stevenson M.M. (2005). Interleukin-15 Enhances Innate and Adaptive Immune Responses to Blood-Stage Malaria Infection in Mice. Infect. Immun..

[211] Dunkelberger J.R., Song W.-C. (2010). Complement and its role in innate and adaptive immune responses. Cell Res..

[212] Wu E.Y., Alexander J.J., Fukui S. (2022). Editorial: The complement system in autoimmunity. Front. Immunol..

[213] Skariah S., Arnaboldi P., Dattwyler R.J., Sultan A.A., Gaylets C., Walwyn O., Mulhall H., Wu X., Dargham S.R., Mordue D.G. (2017). Elimination of *Babesia microti* Is Dependent on Intraerythrocytic Killing and CD4+ T Cells. J. Immunol..

[214] Mahittikorn A., Kwankaew P., Rattaprasert P., Kotepui K.U., Masangkay F.R., Kotepui M. (2022). Elevation of serum interleukin-1β levels as a potential indicator for malarial infection and severe malaria: A meta-analysis. Malar. J..

[215] Chapman W.E., Ward P.A. (1977). *Babesia rodhaini*: Requirement of Complement for Penetration of Human Erythrocytes. Science.

[216] Seinen W., Stegmann T., Kuil H. (1982). Complement does not play a role in promoting *Babesia rodhaini* infections in Balb/C mice. Parasitol. Res..

[217] Levy M.G., Kakoma I., Clabaugh G.C., Ristic M. (1986). Complement does not facilitate in vitro invasion of bovine erythrocytes by *Babesia bovis*. Ann. Trop. Med. Parasitol..

[218] Levy M.G., Kakoma S., Clabaugh G., Ristic M. (1986). Studies on the role of complement in the in vitro invasion of bovine erythrocytes by *Babesia bovis*. Rev. Elev. Med. Vet. Pays Trop..

[219] Kuleš J., Mrljak V., Rafaj R.B., Selanec J., Burchmore R., Eckersall P.D. (2014). Identification of serum biomarkers in dogs naturally infected with *Babesia canis canis* using a proteomic approach. BMC Vet. Res..

[220] Wang X., Ren S., Yang X., Masoudi A., Xue X., Li M., Li H., Zhang X., Wang H., Liu J. (2021). Exploration of Serum Marker Proteins in Mice Induced by *Babesia microti* Infection Using a Quantitative Proteomic Approach. Protein J..

[221] Kuleš J., de Torre-Minguela C., Rafaj R.B., Gotić J., Nižić P., Ceron J., Mrljak V. (2016). Plasma biomarkers of SIRS and MODS associated with canine babesiosis. Res. Vet. Sci..

[222] Zygner W., Gójska-Zygner O., Wędrychowicz H. (2011). Abnormalities in serum proteins in the course of babesiosis in dogs. Bull. Vet. Inst. Pulawy.

[223] Roestenberg M., McCall M., Mollnes T.E., Van Deuren M., Sprong T., Klasen I., Hermsen C.C., Sauerwein R.W., Van Der Ven A. (2007). Complement activation in experimental human malaria infection. Trans. R. Soc. Trop. Med. Hyg..

[224] Behet M.C., Kurtovic L., van Gemert G.-J., Haukes C.M., Siebelink-Stoter R., Graumans W., van de Vegte-Bolmer M.G., Scholzen A., Langereis J.D., Diavatopoulos D.A. (2018). The Complement System Contributes to Functional Antibody-Mediated Responses Induced by Immunization with *Plasmodium falciparum* Malaria Sporozoites. Infect. Immun..

[225] Raballah E., Wilding K., Anyona S.B., Munde E.O., Hurwitz I., Onyango C.O., Ayieko C., Lambert C.G., Schneider K.A., Seidenberg P.D. (2022). Nonsynonymous amino acid changes in the α-chain of complement component 5 influence longitudinal susceptibility to *Plasmodium falciparum* infections and severe malarial anemia in kenyan children. Front. Genet..

[226] Bick R.L. (2002). Disseminated Intravascular Coagulation A Review of Etiology, Pathophysiology, Diagnosis, and Management: Guidelines for Care. Clin. Appl. Thromb. Hemost..

[227] Angchaisuksiri P. (2014). Coagulopathy in malaria. Thromb. Res..

[228] De Gopegui R.R., Peñalba B., Goicoa A., Espada Y., Fidalgo L.E., Espino L. (2007). Clinico-pathological findings and coagulation disorders in 45 cases of canine babesiosis in Spain. Vet. J..

[229] Rafaj R.B., Matijatko V., Kis I., Kučer N., Živičnjak T., Lemo N., Žvorc Z., Brkljačić M., Mrljak V. (2009). Alterations in some blood coagulation parameters in naturally occurring cases of canine babesiosis. Acta Vet. Hung..

[230] Goddard A., Wiinberg B., Schoeman J.P., Kristensen A.T., Kjelgaard-Hansen M. (2013). Mortality in virulent canine babesiosis is associated with a consumptive coagulopathy. Vet. J..

[231] Böhm M., Leisewitz A.L., Thompson P.N., Schoeman J.P. (2006). Capillary and venous *Babesia canis rossi* parasitaemias and their association with outcome of infection and circulatory compromise. Vet. Parasitol..

[232] Jarczak D., Nierhaus A. (2022). Cytokine Storm—Definition, Causes, and Implications. Int. J. Mol. Sci..

[233] Konozy E.H.E., Osman M.E.-F.M., Ghartey-Kwansah G., Abushama H.M. (2022). The striking mimics between COVID-19 and malaria: A review. Front. Immunol..

[234] Alosaimi B., Mubarak A., Hamed M.E., Almutairi A.Z., Alrashed A.A., AlJuryyan A., Enani M., Alenzi F.Q., Alturaiki W. (2021). Complement Anaphylatoxins and Inflammatory Cytokines as Prognostic Markers for COVID-19 Severity and In-Hospital Mortality. Front. Immunol..

[235] Keshari R.S., Silasi-Mansat R., Popescu N.I., Langer M., Chaaban H., Lupu C., Coggeshall M.K., Demarco S., Lupu F. (2015). Complement C5 Inhibition Blocks the Cytokine Storm and Consumptive Coagulopathy by Decreasing Lipopolysaccharide (LPS) Release in *E. coli* Sepsis. Blood.

[236] Ruan C.-C., Gao P.-J. (2019). Role of Complement-Related Inflammation and Vascular Dysfunction in Hypertension. Hypertension.

[237] Strainic M.G., Shevach E.M., An F., Lin F., Medof M.E. (2012). Absence of signaling into CD4+ cells via C3aR and C5aR enables autoinductive TGF-β1 signaling and induction of Foxp3+ regulatory T cells. Nat. Immunol..

[238] Chen J.Y., Cortes C., Ferreira V.P. (2018). Properdin: A multifaceted molecule involved in inflammation and diseases. Mol. Immunol..

[239] Józsi M. (2017). Factor H Family Proteins in Complement Evasion of Microorganisms. Front. Immunol..

[240] Pawluczkowycz A.W., Lindorfer M.A., Waitumbi J.N., Taylor R.P. (2007). Hematin Promotes Complement Alternative Pathway-Mediated Deposition of C3 Activation Fragments on Human Erythrocytes: Potential Implications for the Pathogenesis of Anemia in Malaria. J. Immunol..

[241] Chen J.Y., Galwankar N.S., Emch H.N., Menon S.S., Cortes C., Thurman J.M., Merrill S.A., Brodsky R.A., Ferreira V.P. (2020). Properdin Is a Key Player in Lysis of Red Blood Cells and Complement Activation on Endothelial Cells in Hemolytic Anemias Caused by Complement Dysregulation. Front. Immunol..

[242] David P.H., Hommel M., Miller L.H., Udeinya I.J., Oligino L.D. (1983). Parasite sequestration in *Plasmodium falciparum* malaria: Spleen and antibody modulation of cytoadherence of infected erythrocytes. Proc. Natl. Acad. Sci. USA.

[243] Buffet P.A., Safeukui I., Deplaine G., Brousse V., Prendki V., Thellier M., Turner G.D., Mercereau-Puijalon O. (2011). The pathogenesis of *Plasmodium falciparum* malaria in humans: Insights from splenic physiology. Blood.

[244] Vannier E., Gewurz B.E., Krause P.J. (2008). Human Babesiosis. Infect. Dis. Clin. North Am..

[245] Fraga E., Barreiro J.D., Goicoa A., Espino L., Fraga G., Barreiro A. (2010). Abdominal Ultrasonographic Findings in Dogs Naturally Infected with Babesiosis. Vet. Radiol. Ultrasound.

[246] Schneider D.A., Yan H., Bastos R.G., Johnson W.C., Gavin P.R., Allen A.J., Barrington G.M., Herrmann-Hoesing L.M., Knowles D.P., Goff W.L. (2010). Dynamics of bovine spleen cell populations during the acute response to *Babesia bovis* infection: An immunohistological study. Parasite Immunol..

[247] Leisewitz A.L., Rockett K.A., Gumede B., Jones M., Urban B., Kwiatkowski D.P. (2004). Response of the Splenic Dendritic Cell Population to Malaria Infection. Infect. Immun..

[248] Li S., Goyal B., Cooper J.D., Abdelbaki A., Gupta N., Kumar Y. (2018). Splenic rupture from babesiosis, an emerging concern? A systematic review of current literature. Ticks Tick-Borne Dis..

[249] Bajer A., Rodo A., Welc-Falęciak R., Siński E. (2008). Asymptomatic babesiosis as a cause of splenomegaly and splenectomy in a dog. Med. Weter..

[250] Krogstad D.J., Sutera S.P., Boylan C.W., Gluzman I.Y., Qian Z.F., Rao P.R. (1991). Intraerythrocytic parasites and red cell deformability: *Plasmodium berghei* and *Babesia microti*. Blood Cells.

[251] Brun J.-F., Varlet-Marie E., Myzia J., de Mauverger E.R., Pretorius E. (2021). Metabolic Influences Modulating Erythrocyte Deformability and Eryptosis. Metabolites.

[252] Cunha B.A., Raza M., Schmidt A. (2015). Highly Elevated Serum Ferritin Levels Are a Diagnostic Marker in Babesiosis. Clin. Infect. Dis..

[253] Goff W.L., Johnson W.C., Parish S.M., Barrington G.M., Tuo W., Valdez R.A. (2001). The age-related immunity in cattle to *Babesia bovis* infection involves the rapid induction of interleukin-12, interferon-γ and inducible nitric oxide synthase mRNA expression in the spleen. Parasite Immunol..

[254] Alkadarou T., Musa A., Alkadarou A., Mahfouz M.S., Troye-Blomberg M., Elhassan A.M., Elhassan I.M. (2013). Immunological Characteristics of Hyperreactive Malarial Splenomegaly Syndrome in Sudanese Patients. J. Trop. Med..

[255] Del Portillo H.A., Ferrer M., Brugat T., Martin-Jaular L., Langhorne J., Lacerda M.V.G. (2012). The role of the spleen in malaria. Cell. Microbiol..

[256] Lacerda-Queiroz N., Riteau N., Eastman R.T., Bock K.W., Orandle M.S., Moore I.N., Sher A., Long C.A., Jankovic D., Su X.-Z. (2017). Mechanism of splenic cell death and host mortality in a *Plasmodium yoelii* malaria model. Sci. Rep..

[257] Chauvin A., Moreau E., Bonnet S., Plantard O., Malandrin L. (2009). *Babesia* and its hosts: Adaptation to long-lasting interactions as a way to achieve efficient transmission. Vet. Res..

[258] Bachmann A., Metwally N.G., Allweier J., Cronshagen J., del Pilar Martinez Tauler M., Murk A., Roth L.K., Torabi H., Wu Y., Gutsmann T. (2022). CD36—A Host Receptor Necessary for Malaria Parasites to Establish and Maintain Infection. Microorganisms.

[259] O’Connor R.M., Allred D.R. (2000). Selection of *Babesia bovis*-Infected Erythrocytes for Adhesion to Endothelial Cells Coselects for Altered Variant Erythrocyte Surface Antigen Isoforms. J. Immunol..

[260] Jackson A.P., Otto T.D., Darby A., Ramaprasad A., Xia D., Echaide I.E., Farber M., Gahlot S., Gamble J., Gupta D. (2014). The evolu-tionary dynamics of variant antigen genes in *Babesia* reveal a history of genomic innovation underlying host–parasite in-teraction. Nucleic Acids Res..

[261] Eichenberger R.M., Ramakrishnan C., Russo G., Deplazes P., Hehl A.B. (2017). Genome-wide analysis of gene expression and protein secretion of *Babesia canis* during virulent infection identifies potential pathogenicity factors. Sci. Rep..

[262] Kumar A., Kabra A., Igarashi I., Krause P.J. (2022). Animal models of the immunology and pathogenesis of human babesiosis. Trends Parasitol..

[263] Berendt A.R., McDowall A., Craig A.G., Bates P., Sternberg M., Marsh K., Newbold C., Hogg N. (1992). The binding site on ICAM-1 for plasmodium falciparum-infected erythrocytes overlaps, but is distinct from, the LFA-1-binding site. Cell.

[264] Hubbard A.K., Rothlein R. (2000). Intercellular adhesion molecule-1 (ICAM-1) expression and cell signaling cascades. Free Radic. Biol. Med..

[265] Wung B.S., Ni C.W., Wang D.L. (2005). ICAM-1 induction by TNFα and IL-6 is mediated by distinct pathways via Rac in endothelial cells. J. Biomed. Sci..

[266] El-Assaad F., Wheway J., Mitchell A.J., Lou J., Hunt N.H., Combes V., Grau G.E.R. (2013). Cytoadherence of *Plasmodium berghei*-Infected Red Blood Cells to Murine Brain and Lung Microvascular Endothelial Cells *In Vitro*. Infect. Immun..

[267] Franke-Fayard B., Janse C.J., Cunha-Rodrigues M., Ramesar J., Büscher P., Que I., Löwik C., Voshol P.J., Boer M.A.M.D., van Duinen S.G. (2005). Murine malaria parasite sequestration: CD36 is the major receptor, but cerebral pathology is unlinked to sequestration. Proc. Natl. Acad. Sci. USA.

[268] Nishanth G., Schlüter D. (2019). Blood–Brain Barrier in Cerebral Malaria: Pathogenesis and Therapeutic Intervention. Trends Parasitol..

[269] Cook-Mills J.M. (2002). VCAM-1 signals during lymphocyte migration: Role of reactive oxygen species. Mol. Immunol..

[270] Cook-Mills J.M., Marchese M.E., Abdala-Valencia H. (2011). Vascular Cell Adhesion Molecule-1 Expression and Signaling During Disease: Regulation by Reactive Oxygen Species and Antioxidants. Antioxid. Redox Signal..

[271] Idro R., Jenkins N.E., Newton C.R.J.C. (2005). Pathogenesis, clinical features, and neurological outcome of cerebral malaria. Lancet Neurol..

[272] Sharma L., Shukla G. (2017). Placental Malaria: A New Insight into the Pathophysiology. Front. Med..

[273] Castillo P., Menéndez C., Mayor A., Carrilho C., Ismail M., Lorenzoni C., Machungo F., Osman N., Quintó L., Romagosa C. (2013). Massive *Plasmodium falciparum* visceral sequestration: A cause of maternal death in Africa. Clin. Microbiol. Infect..

[274] Pardini A.D. (2000). The pathology and pathogenesis of canine cerebral babesiosis. Master’s Thesis.

[275] Martin C.A. (2020). Pathology of complicated *Babesia rossi*-associated acute lung injury and respiratory distress syndrome in dogs. Master’s Thesis.

[276] Knackstedt S.L., Georgiadou A., Apel F., Abu-Abed U., Moxon C.A., Cunnington A.J., Raupach B., Cunningham D., Langhorne J., Krüger R. (2019). Neutrophil extracellular traps drive inflammatory pathogenesis in malaria. Sci. Immunol..

[277] Tamarozzi F., Turner J., Pionnier N., Midgley A., Guimaraes A.F., Johnston K.L., Edwards S.W., Taylor M.J. (2016). Wolbachia endosymbionts induce neutrophil extracellular trap formation in human onchocerciasis. Sci. Rep..

[278] Wei R., Li X., Wang X., Wang Y., Zhang X., Zhang N., Wang J., Yang J., Zhang X., Gong P. (2021). Trypanosoma evansi triggered neutrophil extracellular traps formation dependent on myeloperoxidase, neutrophil elastase, and extracellular signal-regulated kinase 1/2 signaling pathways. Vet. Parasitol..

[279] Worku M., Rehrah D., Ismail H., Asiamah E., Adjei-Fremah S. (2021). A Review of the Neutrophil Extracellular Traps (NETs) from Cow, Sheep and Goat Models. Int. J. Mol. Sci..

[280] Morgado F.N., Nascimento M.T., Saraiva E.M., de Oliveira-Ribeiro C., Madeira Mde F., da Costa-Santos M., Vasconcellos E.C., Pimentel M.I., Rosandiski Lyra M., Schubach Ade O. (2015). Are Neutrophil Extracellular Traps Playing a Role in the Parasite Control in Active American Tegumentary Leishmaniasis Lesions?. PLoS ONE.

[281] Kho S., Minigo G., Andries B., Leonardo L., Prayoga P., Poespoprodjo J.R., Kenangalem E., Price R., Woodberry T., Anstey N.M. (2018). Circulating Neutrophil Extracellular Traps and Neutrophil Activation Are Increased in Proportion to Disease Severity in Human Malaria. J. Infect. Dis..

[282] Goggs R., Jeffery U., Levine D.N., Li R.H.L. (2019). Neutrophil-Extracellular Traps, Cell-Free DNA, and Immunothrombosis in Companion Animals: A Review. Vet. Pathol..

[283] Yipp B.G., Kubes P. (2013). NETosis: How vital is it?. Blood.

[284] Debierre-Grockiego F., Moiré N., Arias M.T., Dimier-Poisson I. (2020). Recent Advances in the Roles of Neutrophils in Toxoplasmosis. Trends Parasitol..

[285] Grover S.P., Mackman N. (2018). Neutrophils, NETs, and immunothrombosis. Blood.

[286] Yago T., Liu Z., Ahamed J., McEver R.P. (2018). Cooperative PSGL-1 and CXCR2 signaling in neutrophils promotes deep vein thrombosis in mice. Blood.

[287] Delabranche X., Stiel L., Severac F., Galoisy A.C., Mauvieux L., Zobairi F., Lavigne T., Toti F., Anglès-Cano E., Meziani F. (2017). Evidence of NETosis in Septic Shock-Induced Disseminated Intravascular Coagulation. Shock.

[288] Vardon-Bounes F., Ruiz S., Gratacap M.-P., Garcia C., Payrastre B., Minville V. (2019). Platelets Are Critical Key Players in Sepsis. Int. J. Mol. Sci..

[289] Linkins L.-A., Lapner S.T. (2017). Review of D-dimer testing: Good, Bad, and Ugly. Int. J. Lab. Hematol..

[290] Ni W., Han Y., Zhao J., Cui J., Wang K., Wang R., Liu Y. (2016). Serum soluble urokinase-type plasminogen activator receptor as a biological marker of bacterial infection in adults: A systematic review and meta-analysis. Sci. Rep..

[291] Petersen J.E.V., Kallemose T., Barton K.D., Caspi A., Rasmussen L.J.H. (2020). Soluble urokinase plasminogen activator receptor (suPAR) as a prognostic marker of mortality in healthy, general and patient populations: Protocol for a systematic review and meta-analysis. BMJ Open.

[292] Bui T.M., Wiesolek H.L., Sumagin R. (2020). ICAM-1: A master regulator of cellular responses in inflammation, injury resolution, and tumorigenesis. J. Leukoc. Biol..

[293] Renkonen R., Mennander A., Ustinov J., Mattila P. (1990). Activation of protein kinase C is crucial in the regulation of ICAM-1 expression on endothelial cells by interferon-γ. Int. Immunol..

[294] Renkonen R., Mattila P., Majuri M.L., Paavonen T., Silvennoinen O. (1992). IL-4 decreases IFN-gamma-induced endothelial ICAM-1 expression by a transcriptional mechanism. Scand. J. Immunol..

[295] Kawachi S., Jennings S., Panes J., Cockrell A., Laroux F.S., Gray L., Perry M., van der Heyde H., Balish E., Granger D.N. (2000). Cytokine and endothelial cell adhesion molecule expression in interleukin-10-deficient mice. Am. J. Physiol. Liver Physiol..

